# Reciprocal signaling and direct physical interactions between fibroblasts and breast cancer cells in a 3D environment

**DOI:** 10.1371/journal.pone.0218854

**Published:** 2019-06-24

**Authors:** Deborah J. Wessels, Nikash Pradhan, Yang-Nim Park, Megan A. Klepitsch, Daniel F. Lusche, Karla J. Daniels, Kayla D. Conway, Edward R. Voss, Suchaeta V. Hegde, Thomas P. Conway, David R. Soll

**Affiliations:** Developmental Studies Hybridoma Bank and W.M. Keck Dynamic Image Analysis Facility, Department of Biology, The University of Iowa, Iowa City, Iowa, United States of America; National Centre for Scientific Research-Demokritos, GREECE

## Abstract

Tumorigenic cells undergo cell aggregation and aggregate coalescence in a 3D Matrigel environment. Here, we expanded this 3D platform to assess the interactions of normal human dermal fibroblasts (NHDFs) and human primary mammary fibroblasts (HPMFs) with breast cancer-derived, tumorigenic cells (MDA-MB-231). Medium conditioned by MDA-MB-231 cells activates both types of fibroblasts, imbuing them with the capacity to accelerate the rate of aggregation and coalescence of MDA-MB-231 cells more than four fold. Acceleration is achieved 1) by direct physical interactions with MDA-MB-231 cells, in which activated fibroblasts penetrate the MDA-MB-231/Matrigel 3D environment and function as supporting scaffolds for MDA-MB-231 aggregation and coalescence, and 2) through the release of soluble accelerating factors, including matrix metalloproteinase (MMPs) and, in the case of activated NHDFs, SDF-1α/CXCL12. Fibroblast activation includes changes in morphology, motility, and gene expression. Podoplanin (PDPN) and fibroblast activation protein (FAP) are upregulated by more than nine-fold in activated NHDFs while activated HPMFs upregulate FAP, vimentin, desmin, platelet derived growth factor receptor A and S100A4. Overexpression of PDPN, but not FAP, in NHDF cells in the absence of MDA-MB-231-conditioned medium, activates NHDFs. These results reveal that complex reciprocal signaling between fibroblasts and cancer cells, coupled with their physical interactions, occurs in a highly coordinated fashion that orchestrates aggregation and coalescence, behaviors specific to cancer cells in a 3D environment. These *in vitro* interactions may reflect events involved in early tumorigenesis, particularly in cases of field cancerization, and may represent a new mechanism whereby cancer-associated fibroblasts (CAFs) promote tumor growth.

## Introduction

It is well-established that stromal cells are hijacked by a developing tumor to generate a tumor-specific stroma that, in turn, promotes cancer progression and metastasis [1]. Fibroblasts within the tumor stroma, referred to as “cancer-associated fibroblasts” or CAFs, exhibit a cancer-associated phenotype and have been demonstrated to be major players in cancer progression [2]. Mechanisms whereby CAFs promote tumor progression and metastasis include: 1) extracellular matrix (ECM) remodeling mediated by upregulation of the proteoglycan syndecan I [3, 4] and alterations in collagen composition [5, 6] and 2) secretion of soluble growth factors or cytokines that support cancer cell proliferation, angiogenesis, the epithelial to mesenchymal transition (EMT) [7] and migration [8, 9]. In addition, CAFs may facilitate metastasis by direct contact with cancer cells [9–11]. The relationship between cancer cells and fibroblasts in tumorigenesis is, therefore, reciprocal [12].

Here we have explored reciprocal signaling and physical interactions between breast cancer-derived tumorigenic cells (MDA-MB-231) and normal human dermal fibroblasts (NHDFs) as well as between MDA-MB-231 cells and human primary mammary fibroblasts (HPMFs) in a 3D Matrigel environment in which cancer cells, but not normal cells, aggregate. Aggregates then coalesce to form large aggregates with shapes reflective of their tumor of origin [13, 14]. We found that breast tumor cells release an activation factor(s) that causes changes in both dermal and mammary fibroblast shape and motility, and alterations in gene expression. Although the altered gene expression pattern differs between activated dermal and activated mammary fibroblasts, both types of activated fibroblasts markedly accelerate MDA-MB-231 coalescence relative to unconditioned fibroblasts. Interestingly, activated mammary fibroblasts are even more effective at inducing coalescence of MDA-MB-231than activated NHDFs.

The activated fibroblasts, referred to here as cancer cell conditioned-normal human dermal fibroblasts (CC-NHDFs) or cancer cell conditioned-human primary mammary fibroblasts (CC-HPMFs), are imbued with the capacity to invade the 3D Matrigel environment where they accelerate the rate of MDA-MB-231 cell aggregation and aggregate coalescence. We found that this acceleration is mediated by 1) soluble factors released by activated fibroblasts and 2) by the dynamic participation of CC-NHDFs and CC-HPMFs, which function as scaffolds for MDA-MB-231 aggregation. We further demonstrate that overexpressing podoplanin (PDPN), but not fibroblast activation protein (FAP), in NHDFs in the absence of the soluble activators from cancer cell-conditioned medium, activates fibroblasts, and imbues them with the capacity to accelerate cancer cell aggregation and coalescence.

The functions described here for activated fibroblasts are distinct from the roles typically attributed to CAFs such as promotion of metastasis [10, 15–18] by basement membrane remodeling and stimulation of the epithelial to mesenchymal transition (EMT) [7, 12]. Our data support a model in which CAFs drive coalescence, and by so doing, may promote tumorigenesis, particularly in cases of field cancerization [19, 20].

## Material and methods

### Growth and maintenance of cell lines and primary cells

Normal human dermal fibroblasts (NHDFs) and Fibroblast Growth Medium containing 2% fetal calf serum (FGM), insulin (5μg/mL) and FGF-2 (1ng/mL) were obtained from PromoCell (http://www.promocell.com/) and cells were cultured as specified by the supplier. Human Primary Mammary Fibroblasts (HPMFs), HPMF growth media (HPMF-GM), the Fibroblast Medium Supplement Kit (FBS, hydrocortisone, L-glutamine, FGF and an antibiotic-anti-mycotic solution) and gelatin coating solution were obtained from Cell Biologics (http://www.cellbiologics.net). HPMF cells were cultured in HPMF-GM with the added supplements for 6–7 passages as specified by the supplier. MDA-MB-231 breast cancer cells were obtained from ATCC and cultured for 12–15 passages in MCF media. MCF medium is DMEM/F12 (Life Technologies, Carlsbad, CA) supplemented with 5% horse serum, human recombinant EGF, insulin, hydrocortisone and cholera toxin, all obtained from Sigma Aldrich (St. Louis, MO), and penicillin-streptomycin from Thermo -Fisher (Grand Island, NY) [21]. GFP tagged human dermal fibroblasts (NHDFs-GFP) were obtained from Angio-proteomie (www.angioproteomie.com) and cultured according to the supplier’s directions.

### Activation of fibroblasts by MDA-MB-231-conditioned medium and assay preparations

**Fig 1:**
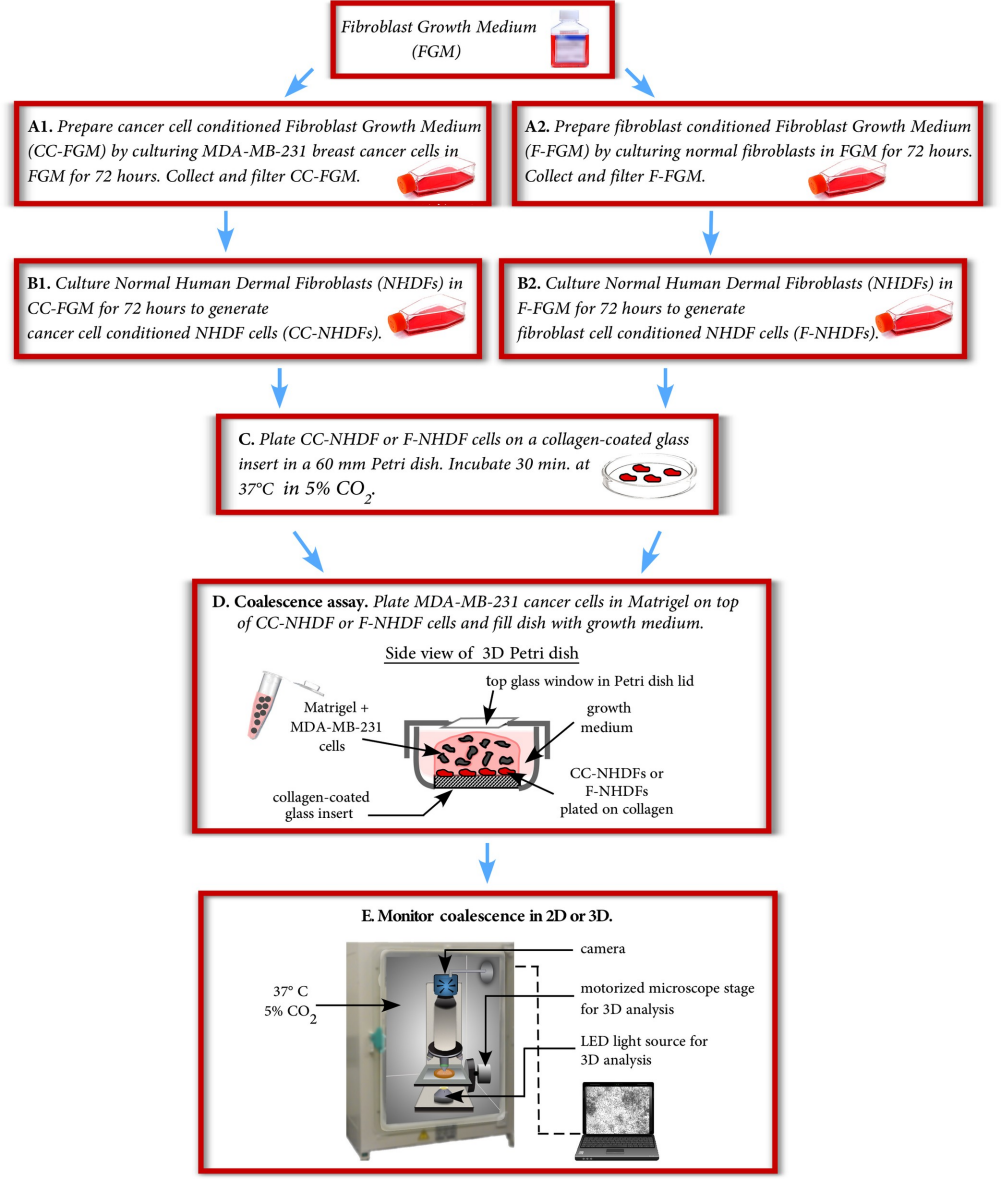
The preparation used to analyze fibroblast cell motility and to compare the effects of F-NHDFs, F-HPMFs, CC-NHDFs and CC-HPMFs on the aggregation and aggregate coalescence of MDA-MB-231 cells.

To generate cancer cell conditioned fibroblast growth media (CC-FGM), MDA-MB-231 cells were grown for 72 hours to 70–80% confluency in 10 mL of FGM containing 2% fetal calf serum (Fig 1A1). The CC-FGM medium was then withdrawn from the culture flask and filtered through a 0.22 μm filter to remove any detached MDA-MB-231cells or cell debris. To generate cancer cell conditioned fibroblasts (CC-NHDFs) (Fig 1B1), NHDFs were grown for 72 hours to 70–80% confluency in the filtered CC-FGM plus 30% fresh FGM. As a control, NHDFs were grown to 70–80% confluency in FGM containing 2% fetal calf serum for 72 hours to generate fibroblast conditioned FGM (F-FGM) (Fig 1A2). The F-FGM was collected and filtered in the same manner as the CC-FGM. Next, NHDFs were grown to 70–80% confluency for 72 hours in the filtered F-FGM to generate fibroblast cell conditioned fibroblasts (F-NHDFs) (Fig 1B2). Cancer cell conditioned HPMFs (CC-HPMFs) and fibroblast cell conditioned HPMFs (F-HPMFs) were generated using this same protocol with HPMF-GM. To determine if results were specific to one formula of media, reciprocal media controls were performed by culturing HPMFs in MCF media [21] and CC-MCF media, again following the protocol illustrated in Fig 1. To determine if growth factors in the growth media were responsible for stimulating the MDA-MB-231 cancer cells to condition the media, minimal conditioned media (CC- minimal MCF) was prepared by culturing MDA-MB-231 cancer cells in MCF media without insulin or EGF and supplemented with 10% serum that had been charcoal stripped to remove growth factors (https://www.atlanta-biologicals.com). Finally, as an additional control, fibroblasts were grown in FGM or HPMF-GM that had not been conditioned by either MDA-MB-231 cells or by fibroblasts.

To prepare samples for 2D analysis of fibroblast motility, the 30 mm glass insert in a 60 mm Petri dish (https://www.cellvis.com/) was pre-coated with Type I human collagen (https://www.advancedbiomatrix.com) according to the supplier’s instructions. Next, a 500 μl aliquot containing 5 x 10^4^ NHDFs in FGM, F-NHDFs in F-FGM, CC-NHDFs in CC-FGM, HPMFs in HPMF-GM, F-HPMFs in F-HPMF-GM, or CC-HPMFs in CC-HPMF-GM was dispersed onto the collagen-coated insert. Cells were allowed to adhere for 30 minutes at 37°C and 5% CO_2_ (Fig 1C) followed by addition of 5 ml of the appropriate media. To assay coalescence in 2D and 3D in the presence of fibroblasts, fibroblasts were first plated on the collagen-coated insert as described above. MDA-MB-231 cells were then harvested and 5x10^5^ cells in 250 μl of medium were mixed with ice-cold Matrigel (Fig 1C) as described elsewhere in detail [13]. Medium was removed from the collagen-coated insert and 750 μl of the cell/Matrigel mixture were gently applied over the fibroblasts (Fig 1D). The Matrigel was allowed to gel by incubating the dishes for 60 minutes at 37°C and 5% CO_2_ and 5 ml of MCF media was then added. 2D and 3D images were acquired on a microscope housed in an incubator at 37°C and 5% CO_2_ (Fig 1E).

### 2D analysis of fibroblast cell shape and motility

Fibroblasts were plated on the collagen-coated insert of a Petri dish as described above. 2D images at 4x or 10x magnification were acquired through an Olympus CK2 microscope housed in an incubator at 37°C and 5% CO_2_. Illumination and acquisition were synchronized using Fire-I software (www.unibrain.com/products/fire-i-software/). A series of JPEG images were acquired every 45 seconds with a XCD-V50 camera (Sony, San Diego, CA). The images were imported into J3D-DIAS4.2 [13, 14, 22–24] and compiled into JDIAS movie format for motion and shape analysis. Cells were outlined at 7.5 minute intervals for 6 hours. Stacked perimeter plots were generated and length, width, perimeter, area and instantaneous velocity calculated as described previously [25]. J3D-DIAS4.2 calculates persistence as the ratio of net to total path length at 5 frame intervals and averaged over the total path length.

### 2D and 3D analysis of MDA-MB-231 aggregation and aggregate coalescence in the presence of NHDF, F-NHDF, F-HPMF, CC-NHDF and CC-HPMF cells

NHDFs, F-NHDF, HPMFs, F-HPMFs, CC-NHDFs or CC-HPMFs were plated onto the collagen-coated insert and overlayed with a Matrigel/MDA-MB-231 cell suspension as described above. 2D images at 4X and 10X magnification were acquired through an Olympus CK2 microscope housed in an incubator at 37°C and 5% CO_2_. For 3D analyses of coalescence with DIC optics, a lid with a glass insert was used to cover the dish. 3D cultures were imaged through a 20x objective using Differential Interference Contrast (DIC) optics on a Zeiss Axioplan 2 microscope with a motor-driven stage synchronized to a Zeiss AxioCam MRc5 IEEE 1394 color CCD camera and an LED light source. The microscope was housed in an incubator at 37°in 5% CO_2_. A z-series of optical sections was acquired through the preparation at 10 μm increments and this process repeated every 10 minutes for up to 5 days. The z-series was imported into J3D-DIAS4.2 and saved in movie format for 3D reconstructions, as previously described [13, 14, 22, 23].

### Immunostaining

Fibroblasts were plated on the collagen-coated insert (described above) and incubated for 60 minutes at 37° C in 5% CO_2_ before addition of 5 mL of media to the dish. After 24 hours, the preparation was rinsed with PBS and immunostained as described previously [14]. Cells were stained with the anti-vimentin monoclonal antibody (mAb) AMF-17b (Developmental Studies Hybridoma Bank, (DSHB) http://dshb.biology.uiowa.edu) diluted 1:10 in PBS, or the rat anti-human podoplanin mAb NZ-1 (AngiobioCo., San Diego, CA) diluted 1:200 in PBS. The AMF-17b preparation was stained with the secondary antibody AlexaFluor 488 conjugated goat anti-mouse IgG (Jackson ImmunoResearch), and the NZ-1 preparation with the secondary antibody AlexaFluor 488 conjugated goat anti-rat IgG (Jackson ImmunoResearch).

### qRT-PCR

For RNA preparations, conditioned and unconditioned fibroblast cells were washed in PBS, resuspended in RNAlater solution (Ambion, Life technologies, Carlsbad, CA, USA) and incubated for 1 hour at 4°C. RNA was extracted using the RNeasy Mini kit (QIAGEN). The TURBO DNA-free kit (Ambion, Life technologies, Carlsbad, CA, USA) was employed to remove DNA. The quality of the RNA was confirmed with the Experion RNA StdSens and HighSens Analysis Kit (Bio-Rad) that assigned an RNA Quality Index (RQI) >9.5 for our samples, indicating little to no RNA degradation had occurred. To generate cDNA from the RNA samples, the iScript cDNA Synthesis kit (Bio-Rad) was used, as recommended by the manufacturer. qRT-PCR assays were performed with LightCycler 480 SYBR Green I Master mix (Roche) using 50 ng of the cDNA. The expression levels of the genes of interest were quantified using a Roche LightCycler480 real-time PCR detection system with SYBR green. The qRT-PCR assay for each of the tested genes was repeated three times, each in triplicate. The relative expression level of the genes was normalized to that of *GAPDH*. Primer pairs used for qRT-PCR are listed in S1 Table.

### Western blot analysis of cell lysates and media

Western analysis of protein from cell lysates was performed as previously described [26]. Rat anti-podoplanin NZ-1 (AngiobioCo., San Diego, CA) and mouse anti-FAP (Santa Cruz Biotechnology, INC) were employed as primary antibodies. Mouse anti-β-tubulin mAb E7 (DSHB; http://dshb.biology.uiowa.edu) was used as a loading control. To assess the release of SDF-1α/CXCL12, media were concentrated with Amicon Ultra 3K centrifugal filter devices (Merck Millipore Ltd.) according to the supplier’s protocol. Recombinant human SDF-1α/CXCL12 (R&D Systems; https://www.rndsystems.com) was used as a positive control. Rabbit anti-human SDF-1α/CXCL12 antibody (Thermo Fisher Scientific) was used to detect the cytokine. IRDye 800-conjugated goat anti-rat, goat anti-mouse or goat anti-rabbit antibody (Li-Cor Biosciences, Lincoln, NE) were used as secondary antibodies. Odyssey scanner and software were used for detection and quantification of immunoblots (Li-Cor Biosciences).

### Transwell assay with NHDF cells

A Corning Incorporated Transwell plate with 24 mm inserts, the latter constructed from 0.4 μm pore membranes (https://www.corning.com/worldwide/en.html), was used to test whether conditioned fibroblasts released a soluble factor capable of traversing the membrane to affect MDA-MB-231 cell behavior in the absence of fibroblast/cancer cell-cell contact. The plate well was first pre-coated with 100 μl of Matrigel. MDA-MB-231 cells were diluted to 1×10^6^ cells per ml in MCF medium, chilled on ice, and a 500 μl aliquot mixed with 1 ml of ice-cold Matrigel. The chilled MDA-MB-231/Matrigel mixture was then inoculated into the pre-coated well. F-NHDF or CC-NHDF cultures, containing 4×10^4^ cells, were suspended in 400 μl of F-FGM or CC-FGM medium, respectively, and spread on the insert filter over an empty well. The plate was then allowed to incubate for 30 minutes at 37°C to allow for gelation of the Matrigel as well as attachment of the fibroblasts to the insert filter. Media were removed from the inserts and the inserts transferred on to wells containing the MDA-MB-231 cell/Matrigel mixture. The transwell preparation was incubated for 60 minutes at 37°C and 5% CO_2_ to allow gelation of the Matrigel, followed by addition of 2.5 ml of MCF medium to each well. Images were acquired daily for four days using the 10X objective of a Zeiss Axiovert 100 microscope. To confirm that fibroblasts could not migrate through the insert filter, the transwell assay was performed using NHDF cells in the absence of MDA-MB-231 cells and the bottom well examined microscopically.

### Development of NHDF overexpression strains

To overexpress human *PDPN* and *FAP* genes transiently in NHDFs, we constructed plasmids pPDPN-C3 and pFAP-C3. To construct the overexpression plasmids, the *PDPN* and *FAP* genes were amplified by PCR from the plasmids containing human *PDPN* cDNA (http://dnasu.org/DNASU/GetCloneDetail.do?cloneid=443900) and human *FAP* cDNA (http://dnasu.org/DNASU/GetCloneDetail.do?cloneid=43440) with the primer pairs PDPN-1/-2 and FAP-1/-2, respectively. The PCR amplified coding regions of the genes were cloned under the control of CMV promoter in the plasmid pEGFP-C3 (BD Bioscienes Clonetech, Mountain View, CA, USA) by enzyme restriction with AgeI and SalI and followed by ligation. To generate a control expression plasmid pmCherry-C3, the *EGFP* coding region in the plasmid pEGFP-C3 was swapped with the *mCherry* gene that was amplified from a plasmid with *mCherry* by PCR with primer pair mCherry-1/-2. Primer pairs used for generating the overexpression and control plasmids are listed in S1 Table. All sequences of the genes were verified after cloning. To generate *PDPN* and *FAP* overexpression strains, NHDF-PDPN^oe^ and NHDF-FAP^oe^ transfections of the plasmids into NHDF cells were performed using FuGENE transfection reagent (www.promega.com) according to the supplier’s specifications and confirmed by positive expression of mCherry.

### Treatment of F-NHDFs and CC-NHDFs with anti-PDPN mAb

mAb NZ-1, described above, was affinity purified using a Protein G HP spin trap column (GE Healthcare, Cat.#28903134) and concentrated using an ultra-centrifugal filter (EMD Millipore, Cat.# UFC905024) [27]. The concentration was measured by a Nanodrop 1000 spectrophotometer (ThermoScientific) and 0.67 μg added to 4x10^4^ F-NHDFs or CC-NHDFs in 200 μl of medium. The preparations were incubated at room temperature for 20 minutes. The contents of each tube were then divided equally between two collagen-coated wells. As controls, mAb in 200 μl PBS was processed similarly in the absence of cells. The plates were then incubated for 30 minutes at 5% CO_2_ at 37°C. An MDA-MB-231/Matrigel mixture was then cast over the fibroblast cell layer as described above. Images were acquired daily for four days using the 10X objective of a Zeiss Axiovert 100 microscope.

### MMP Gelatinase Zymography and treatment with MMP inhibitor

Sterile-filtered media were diluted 1:1 with zymogram sample buffer (BioRad) and 20 μl loaded into each well of a Tris-Glycine Novex 10% zymogram protein gel copolymerized with 0.1% gelatin (Invitrogen). SDS-Page gels were run at 118V constant voltage for 90 minutes. Gelatinase activity was detected following the manufacturer’s protocol. Images of the gel demonstrating cleared regions of enzymatic activity were captured using a light box and Nikon CoolPix camera. Digital images were quantitated using ImageJ software [28]. NHDFs were cultured in CC-FGM with or without 50 μM of the MMP inhibitor GM6001 (http://www.emdmillipore.com) for 72 hours.

### Treatment of MDA-MB-231 cells with SDF-1-α/CXCL12, anti- SDF-1-α/CXCL12 mAb and IL-6

Recombinant SDF-1α/CXCL12 and human/mouse SDF-1α /CXCL12 mAb were obtained from R&D Systems (https://www.rndsystems.com) and reconstituted according to the supplier’s specifications. MDA-MB-231 cells were grown for 24 hours in 10 ml of MCF media supplemented with 3 μg of the reconstituted protein or supplemented with 3 μg of the protein plus 25 μg of the anti-SDF-1α mAb. To test the effect of IL-6 on MDA-MB-231s, cells were grown for 24 hours in MCF medium supplemented with 50 ng/mL of recombinant IL-6 (rIL-6, www.peprotech.co). To test the effect of rIL-6 on MDA-MB-231 cells, the rIL-6 treated MDA-MB-231 cells were embedded in 3D Matrigel as previously described [14]. Images of 10 fields were taken at 24 hour intervals using a 4X objective.

## Results

### Breast cancer cell-conditioned medium activates NHDFs and alters gene expression

**Fig 2:**
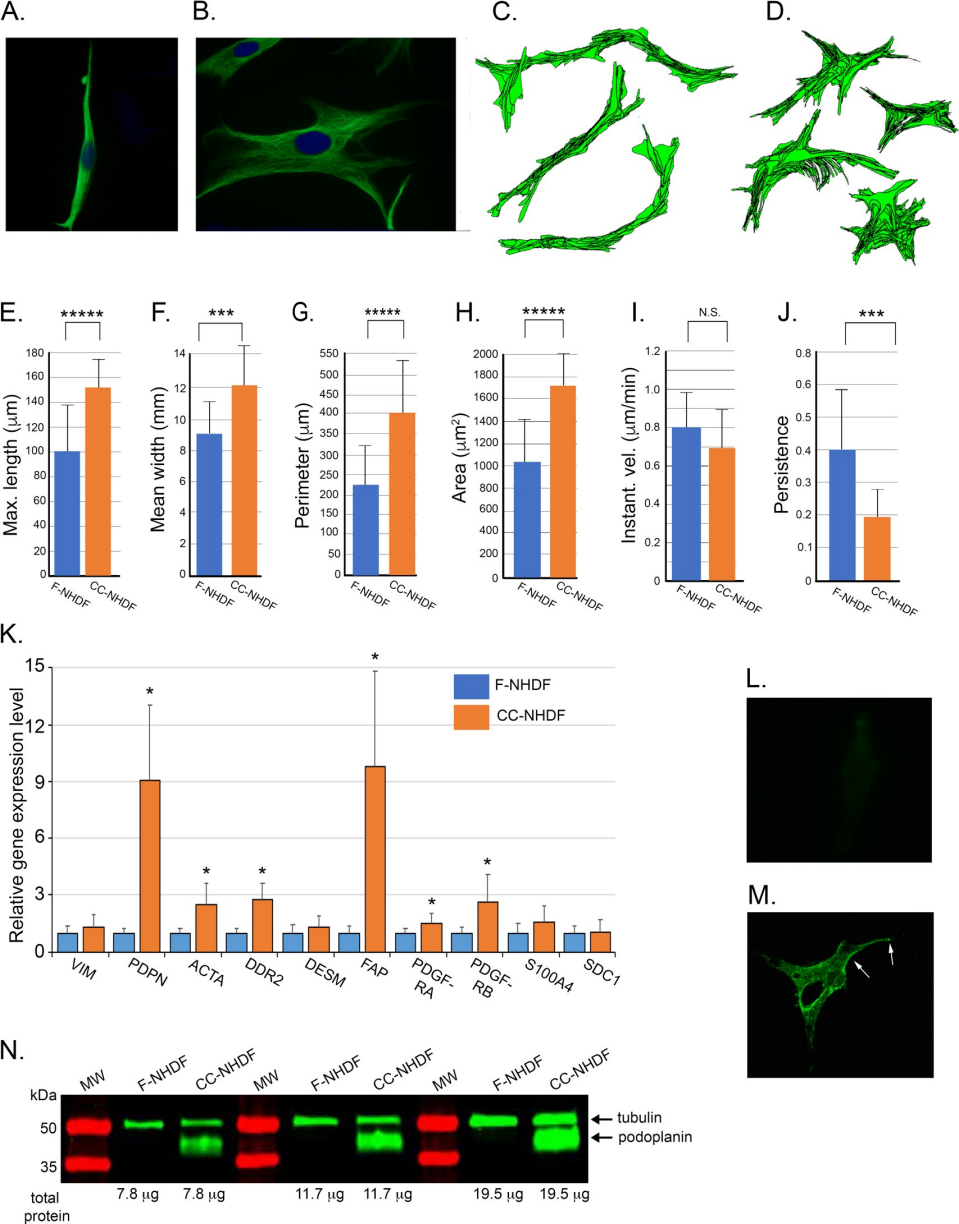
Medium conditioned by the breast tumor-derived cell line MDA-MB-231 activates NHDFs affecting polarity, cellular translocation, morphology and gene expression. A, B. F-NHDFs and CC-NHDFs, respectively, stained for vimentin (green) to visualize cell shape, and with DAPI (blue) to visualize nuclei. C-D. Motility tracks at 7.5 minute intervals of F-NHDFs and CC-NHDFs, respectively, generated by J3D-DIAS4.2 software that automatically detects cell perimeters and computes tracks. E- J. J3D-DIAS4.2 computations of length, width, perimeter, area, instantaneous velocity and the persistence of translocation. The means and error bar (standard deviations) are presented in all panels for an N = 50. ***** indicates significance of p<0.00005, *** indicates significance p<0.0005, N.S indicates not significant. K. qRT-PCR analyses of the transcript levels of ten genes associated with fibroblast activation. Means and error bars (standard deviations) are presented for N = 3. Asterisk (*) indicates significance of p <0.05. L,M. F-NHDFs and CC-NHDFs, respectively, stained with anti-PDPN mAb NZ-1. Arrows denote punctate plasma membrane staining. N. Western blot of F-NHDF and CC-NHDF lysates probed with anti-PDPN mAb NZ-1 and the anti-tubulin mAb E7.

To determine if treatment of fibroblasts with media conditioned by MDA-MB-231 cells altered the shape and motility of the fibroblasts, NHDF cells were treated with cancer cell conditioned or fibroblast cell conditioned media as described in the Methods. A comparison of F-NHDFs and CC-NHDFs, fixed on a collagen substrate and stained with the anti-vimentin mAb AMF-1b, revealed markedly different morphologies (Fig 2 A and 2B). F-NHDFs were spindle shaped and bipolar (Fig 2A), whereas CC-NHDFs were spread and multipolar (Fig 2B). On a collagen-coated 2D substrate, cellular translocation tracks of F-NHDFs were long and persistent, (Fig 2C), while tracks of CC-NHDFs were random and less persistent (Fig 2D). Morphometric measurements revealed that CC-NHDFs were on average significantly longer (Fig 2E), presumably due to the numerous elongated lamellipodia and filopodia, while F-NHDFs were significantly narrower (Fig 2F), and possessed on average a much smaller perimeter (Fig 2G) and a significantly smaller area (Fig 2H), than CC-NHDFs. Instantaneous velocity, computed without directional bias [25], was similar for CC-NHDFs and F-NHDFs (Fig 2I), but the computed persistence of translocation was significantly higher for F-NHDFs than CC-NHDFs (Fig 2J). These results indicate that factors released by MDA-MB-231 cells alter both the morphology and behavior of dermal fibroblasts.

Next, to determine if factors released by MDA-MB-231 cells altered gene expression in CC-NHDFs, expression of ten genes previously demonstrated to be involved in fibroblast activation and/or motility [29] was assessed by quantitative reverse transcriptase-polymerase chain reaction (qRT-PCR). The genes included vimentin (VIM), PDPN, α-smooth muscle actin (ACTA), the tyrosine kinase discoidin domain-containing receptor 2 (DDR2), desmin (DES), FAP, platelet-derived growth factor receptor A (PDGFRA), platelet-derived growth factor receptor B (PDGFRB), Ca^++^ binding protein (FSP1/S100A4), and syndecan1 (SDC1). Five of the genes, PDPN, ACTA, DDR2, FAP and PDGFRB were up-regulated in CC-NHDFs by more than two-fold (Fig 2K). PDPN was upregulated nine-fold and FAP ten-fold (Fig 2K). F-NHDF cells did not stain with the anti-podoplanin mAb (Fig 2L), but CC-NHDF cells stained intensely (Fig 2M, arrows). Western blot analysis supported the fluorescence data, demonstrating that PDPN was expressed in CC-NHDFs, but not in F-NHDFs (Fig 2N). Western blot analyses of FAP revealed that it was present in F-NHDFs, and the level increased several fold in CC-NHDFs (S1A Fig). Therefore, factors released by MDA-MB-231 cells induce changes in NHDF gene expression consistent with fibroblast activation.

### CC-NHDF cells accelerate MDA-MB-231 cell aggregation and aggregate coalescence

**Fig 3:**
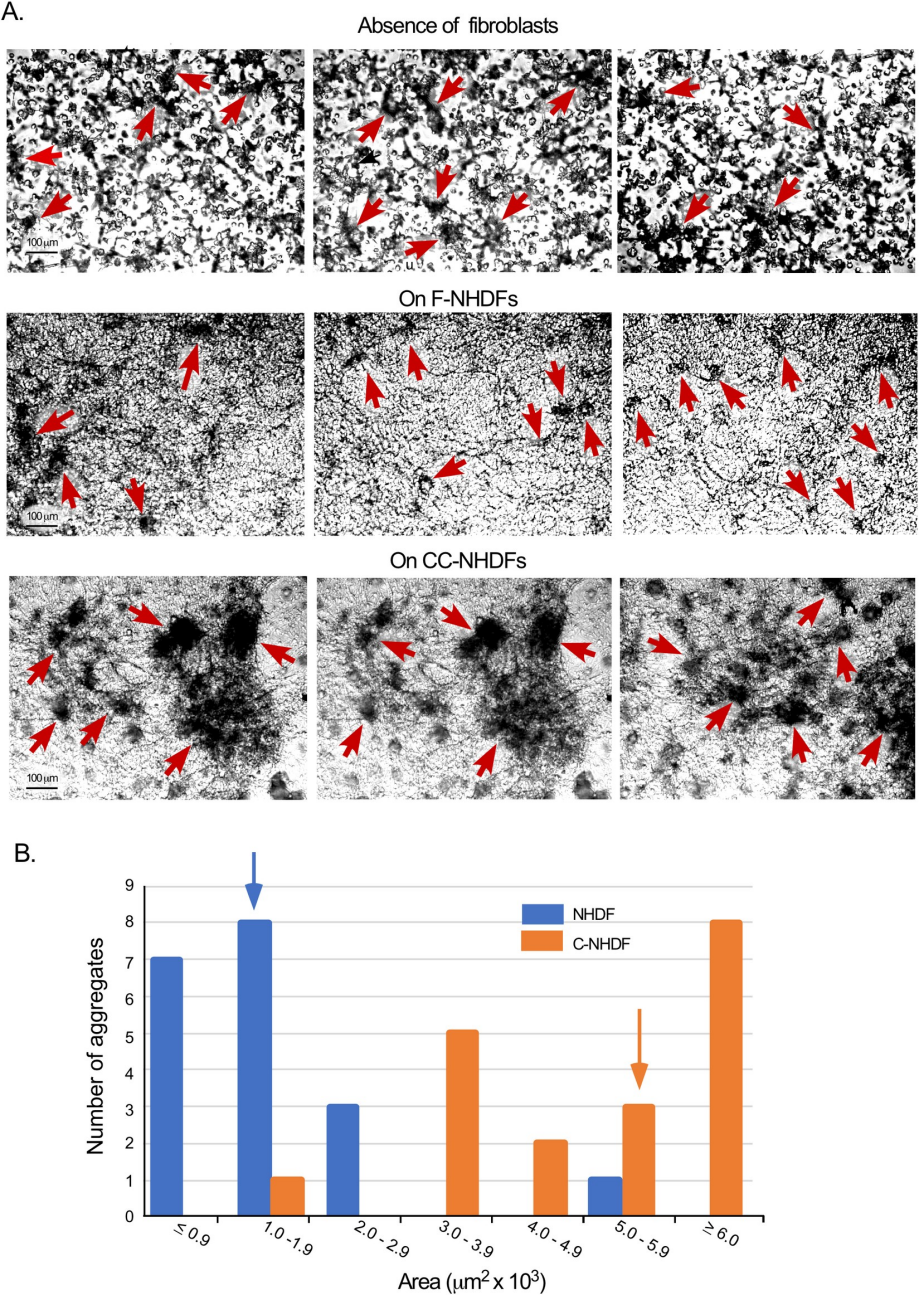
CC-NHDFs but not F-NHDFs accelerate MDA-MB-231 cell aggregation and aggregate coalescence in a 3D environment. A. Representative fields of aggregating MDA-MB-231 cells in which the MDA-MB-231/Matrigel phase is cast over the substrate in the absence of fibroblasts, over F-NHDF cells on collagen and over CC-NHDF cells on collagen. Red arrows indicate MDA-MB-231 aggregates. B. Measurements of aggregate areas at 72 hours in cultures in which the MDA-MB-231/Matrigel phase was cast over F-NHDF cells (blue) or CC-NHDF cells (orange). The median area of each population is noted as a blue or orange arrow, respectively. The difference between MDA-MB-231 aggregate areas was significantly greater (p< 0.00001) in the presence of CC-NHDF when compared to F-NHDFs.

In the experimental protocol employed, in the absence of a fibroblast substratum, cell aggregation and aggregate coalescence (hereafter referred to as “aggregation and coalescence”) occurred slowly over 192 hours. At 72 hours, cells had moved through the gel, forming small aggregates (Fig 3A, arrows). Aggregation and coalescence continued so that by 144 hours, the great majority of cells in the field resided in a few large aggregates, and the surrounding Matrigel was depleted of single cells as previously described [13, 22]. When MDA-MB-231 cells were cast over F-NHDFs, the rates of aggregation and coalescence were similar (Fig 3A, arrows). When MDA-MB-231 cells were cast in Matrigel over CC-NHDFs, aggregation and coalescence occurred at a dramatically accelerated rate, forming large aggregates by 72 hours (Fig 3A, arrows), at least four-fold greater than preparations cast over F-NHDF cells. Quantitative analysis of the size of aggregates in 10 different fields of duplicate 72 hour cultures confirmed these differences (Fig 3B). The median area of MDA-MB-231 cell aggregates formed over CC-NHDFs was more than four-fold larger than those formed over F-NHDFs (Fig 3B).

### CC-NHDFs attract MDA-MB-231 cells and function as scaffolds in the 3D model

**Fig 4:**
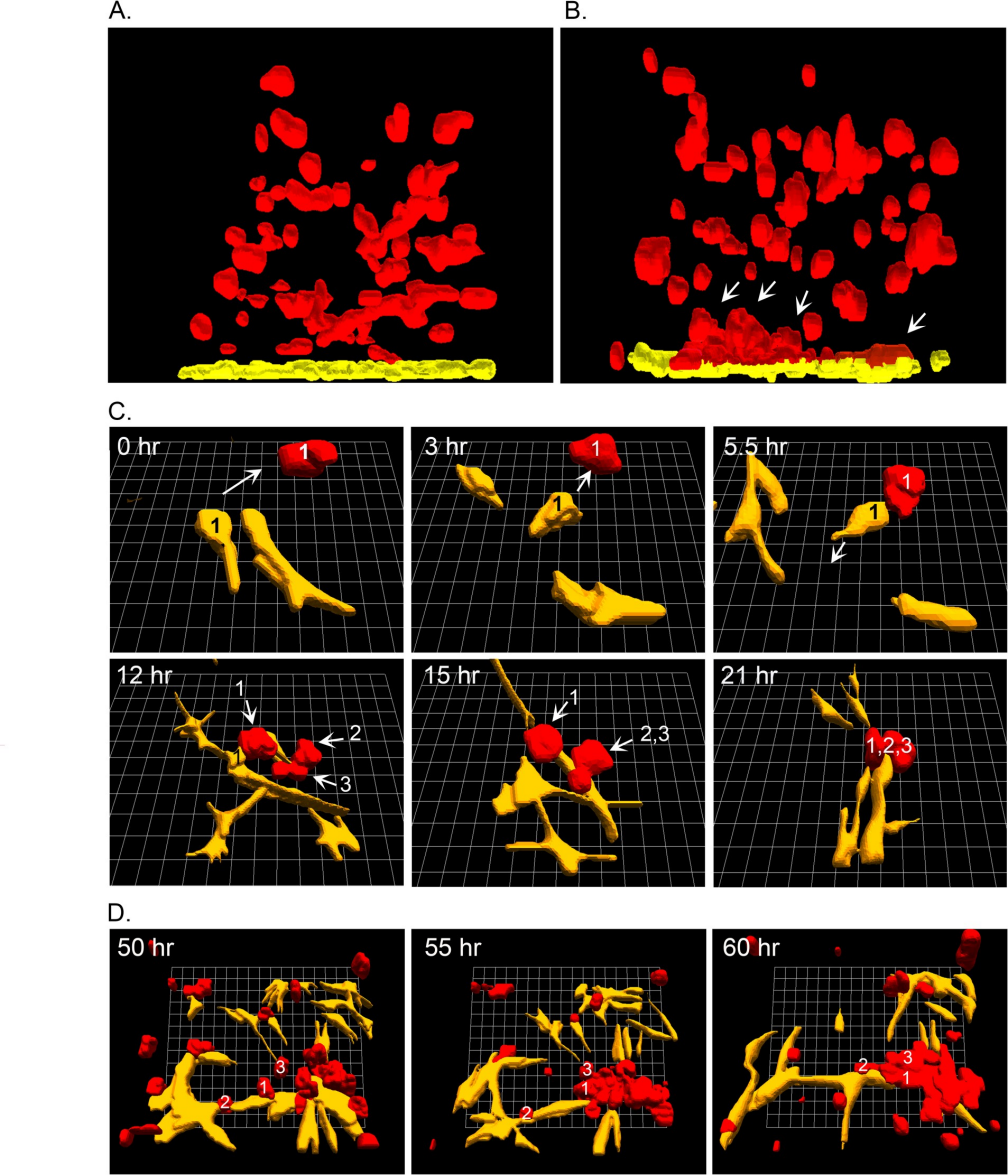
CC-NHDF cells attract MDA-MB-231 cells and function as scaffolds to accelerate aggregation and coalescence of cancer cells. A, B. Side views of 3D reconstructions of MDA-MB-231 cells (red) in Matrigel cast over NHDF cells (yellow) on collagen or CC-NHDF cells (yellow) on collagen, respectively, after 24 hours of incubation. Arrows point to MDA-MB-231 aggregates forming at or near the collagen/Matrigel interface and in physical contact with CC-NHDFs. C. 3D of CC-NHDFs (yellow) and MDA-MB-231 cells (red) over a 21 hour period, with both cell types tracked by analyzing the optical section stacks at 10 minute intervals. D. 3D reconstructions of CC-NHDFs (yellow) and MDA-MB-231 cells (red) between 50 and 60 hours of incubation. Reconstructions were performed with J3D-DIAS4.2 software [13, 14, 23, 30].

2D imaging suggested that accelerated aggregation and coalescence was more pronounced in the Matrigel region closest to the CC-NHDF substratum. To explore this observation, we used the 3D reconstruction software J3D-DIAS4.2 [13, 23, 30]. In Fig 4A and 4B, side views of reconstructions are presented of a MDA-MB-231/Matrigel preparation cast over F-NHDF and CC-NHDF substrates. Fibroblasts are color-coded yellow and MDA-MB-231 cells and aggregates color-coded red. The MDA-MB-231 cells and small aggregates over the F-NHDF substratum remained dispersed throughout the 3D Matrigel matrix after 24 hours (Fig 4A), whereas MDA-MB-231 cells and small aggregates accumulated in the Matrigel region closest to the interface with the CC-NHDF substratum (Fig 4B, white arrows). The F-NHDF or CC-NHDF cells were distinguishable from the MDA-MB-231 cells over time by visually monitoring the optical sections collected every 10 minutes.

To further investigate the interactions between CC-NHDFs and MDA-MB-231 cells in the 3D Matrigel environment, we reconstructed single cells and clusters of cells using J3D-DIAS4.2 [13, 14, 23, 30]. In the first reconstructions in Fig 4C, a CC-NHDF cell (yellow, 1), at the CC-NHDF-MDA-MB-231/Matrigel interface at 0 hours moved in a directed fashion (arrow) towards an MDA-MB-231 cell (red, 1) between 0 and 3 hours. At 5.5 hours, the CC-NHDF contacted MDA-MB-231 cell 1, changed direction, and migrated towards a developing aggregate, with MDA-MB-231 cell 1 in tow (Fig 4C). During that same time period, two additional MDA-MB-231 cells, (red 2 and 3), in contact with another CC-NHDF cell, moved in the direction of CC-NHDF cell 1, and by 21 hours, MDA-MB-231 cells 1, 2 and 3 had aggregated around several CC-NHDF cells, including CC-NHDF cell 1 (Fig 4C). In a second reconstruction in a later time period, shown in Fig 4D, MDA-MB-231 cells (red) aggregated using CC-NHDF cells (yellow), as scaffolds. These behaviors were observed in additional regions optically sectioned in a similar fashion in multiple preparations.

### CC-NHDFs penetrate the MDA-MB-231/Matrigel overlay

**Fig 5:**
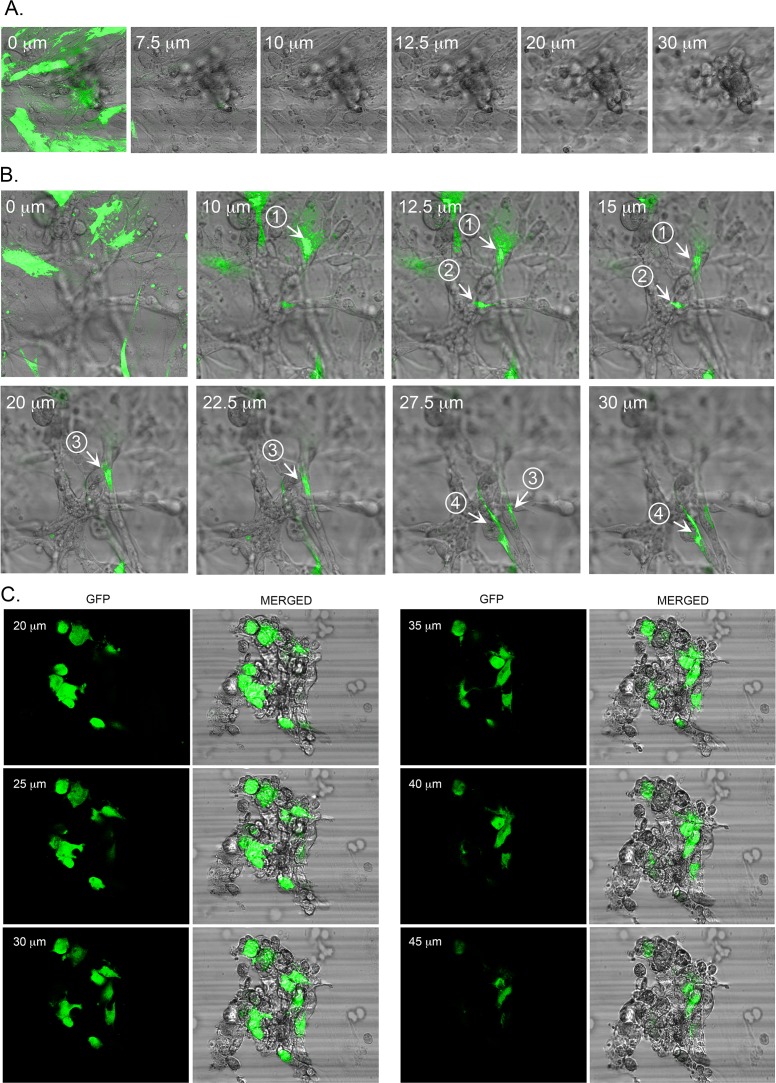
CC-NHDFs act as scaffolds and penetrate MDA-MB-231 aggregates to support and facilitate aggregation. A. F-NHDF-GFP cells in the basal layer do not penetrate the MDA-MB-231/Matrigel overlay after 72 hours of incubation. B. CC-NHDF-GFP cells in the basal layer penetrate through at least 30 μm of the MB-231/Matrigel upper phase after 72 hours of incubation. Fluorescent images were combined with phase contrast images at ascending depths through the upper phase. Cells or their projections are numbered. C. Optical sections of an aggregate at 96 hours of culture formed in the Matrigel phase of a preparation in which CC-NHDF-GFP cells were plated on the collagen substratum. The fluorescent images of the CC-NHDFs and the phase DIC images of all the cells are merged for each optical section.

In examining optical sections, it also appeared that CC-NHDFs, but not F-NHDFs, penetrated the MDA-MB-231/Matrigel overlay. To explore this observation, the MDA-MB-231/Matrigel mixtures were cast over NHDF-GFP cells treated with either fibroblast conditioned medium (“F-NHDF-GFP cells”) or MDA-MB-231-conditioned medium (“CC-NHDF-GFP cells”). DIC and fluorescent images were obtained at different distances through the MDA-MB-231/Matrigel phase after 72 hours of incubation. No F-NHDF-GFP cells were observed through 5 to 45 μm of the Matrigel phase (Fig 5A). However, CC-NHDF-GFP cells, were observed extending at least 30 μm into the MDA-MB-231/Matrigel phase (Fig 5B). After 96 hours, CC-NHDF-GFP cells (green) were enmeshed in the MDA-MB-231 cell aggregates within the Matrigel overlay (Fig 5C).

A quantitative analysis was performed to verify that CC-NHDF cells had a greater propensity to invade the Matrigel and incorporate into MDA-MB-231 aggregates than F-NHDF cells. An examination of 10 independent aggregates at 172 hours in preparations containing F-NHDF-GFP cells revealed F-NHDF-GFP cells in 23% of MDA-MB-231 aggregates. The average number of F-NHDF-GFP cells within the 23% of aggregates containing them was 3 ± 1.8. The highest level in the 150 μm z-series at which F-NHDF-GFP cells appeared in the aggregates was 33.6 μm ± 14.3. In contrast, 100% of the 10 aggregates examined in the CC-NHDF-GFP preparations contained CC-NHDF-GFP with an average number of 54 ± 15 CC-NHDF-GFP cells per aggregate (p<0.005). The average highest level in which CC-NHDF-GFP cells appeared in the aggregates was 100 μm ± 28.2 (p<0.0005).

### The role of PDPN

**Fig 6:**
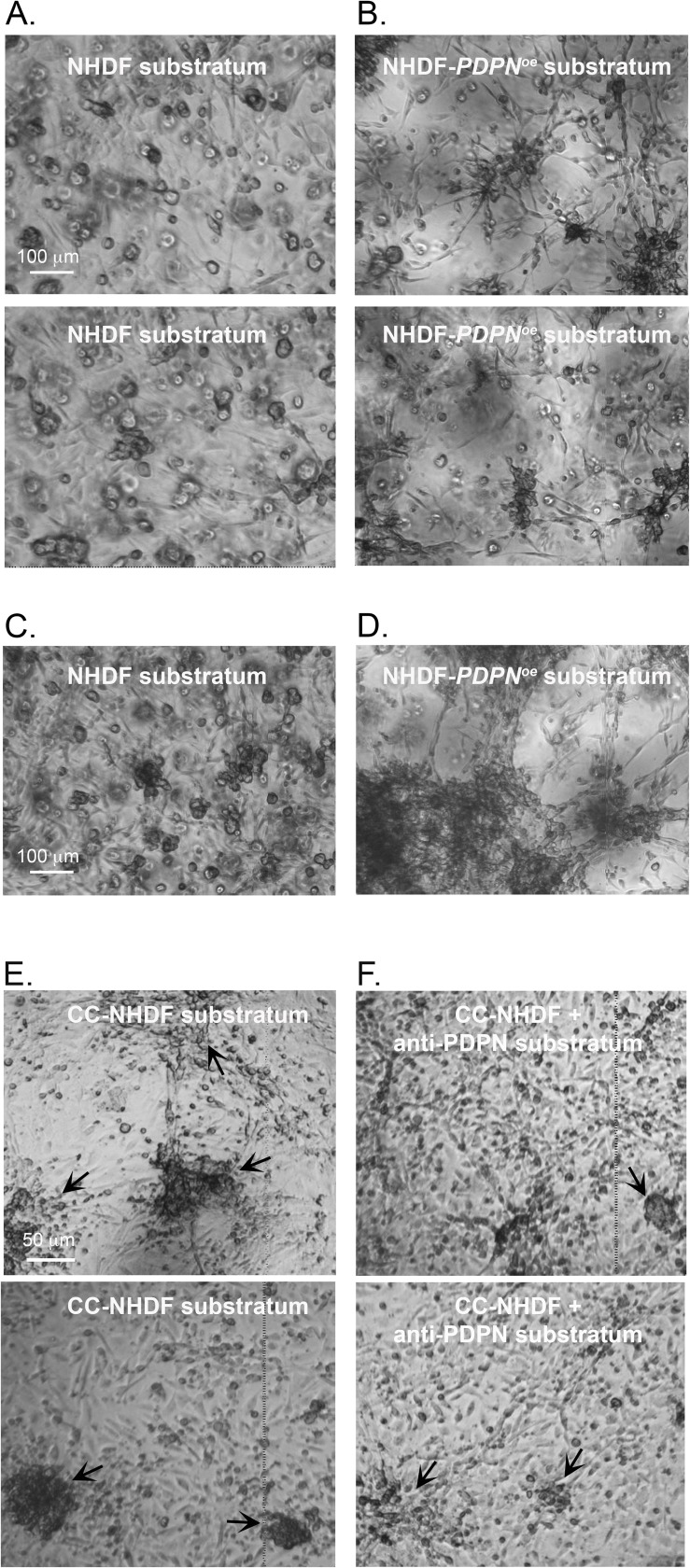
Overexpression of podoplanin imbues NHDFs not activated with MDA-MB-231 conditioned medium with the capacity to accelerate aggregation and coalescence, and pretreatment of CC-NHDFs with anti-podoplanin mAb blocks its capacity to accelerate. A. Aggregation and aggregate coalescence of MDA-MB-231 preparations after 24 hours in which the substratum was NHDF cells on collagen. B. Aggregation and aggregate coalescence of MDA-MB-231 preparations after 24 hours in which the substratum was NHDF-*PDPN*^*œ*^ cells on collagen. C, D. Aggregation and coalescence of MDA-MB-231 at 72 hours, in which the substratum was either NHDF (C) or NHDF-*PDPN*^*œ*^ cells (D) on collagen. E, F. Aggregation and aggregate coalescence of MDA-MB-231 after 72 hours in which the substratum was either untreated CC-NHDFs (E) or CC-NHDFs pretreated with the anti-podoplanin mAb NZ-1 (F).

Because PDPN and FAP were upregulated at the transcript level more than nine-fold in CC-NHDFs, we assessed whether upregulation of either played a role in the acquisition of the capacity of CC-NHDFs to accelerate aggregation and coalescence of MDA-MB-231 cells. To that end, we generated the *PDPN* and *FAP* overexpression strains NHDF-*PDPN*^*œ*^ and NHDF-*FAP*^*œ*^. We then tested whether overexpression of either resulted in activation of NHDF cells, thereby imbuing them with the capacity to accelerate MDA-MB-231 cell aggregation and coalescence in a 3D environment. After 24 hours, there was minimal aggregation of the MDA-MB-231 cells in Matrigel cast over a control NHDF substratum (Fig 6A). In marked contrast, after 24 hours, aggregation and coalescence of MDA-MB-231 cells in Matrigel cast over a NHDF-*PDPN*^*œ*^ substratum was accelerated (Fig 6B). After 72 hours, while MDA-MB-231 cells over the NHDF cells had formed medium-sized aggregates (Fig 6C), the majority of MDA-MB-231 cells over NHDF-*PDPN*^*œ*^ cells had coalesced into large aggregates (Fig 6D). Overexpressing *FAP* in NHDF cells, however, did not imbue them with the capacity to accelerate (S1B Fig). To further explore the role of PDPN, we tested whether anti-podoplanin mAb NZ-1 affected acceleration. Compared to the coalescence on a substratum of CC-NHDF cells (Fig 6E), NZ-1 reduced the rate of aggregation and coalescence (Fig 6F), reinforcing the conclusion that PDPN plays a central role in the capacity of CC-NHDF cells to accelerate aggregation and coalescence of MDA-MB-231 cells in a 3D environment.

### Matrix metalloproteinases (MMPs) and SDF-1α/CXCL12 accelerate aggregation and coalescence

**Fig 7:**
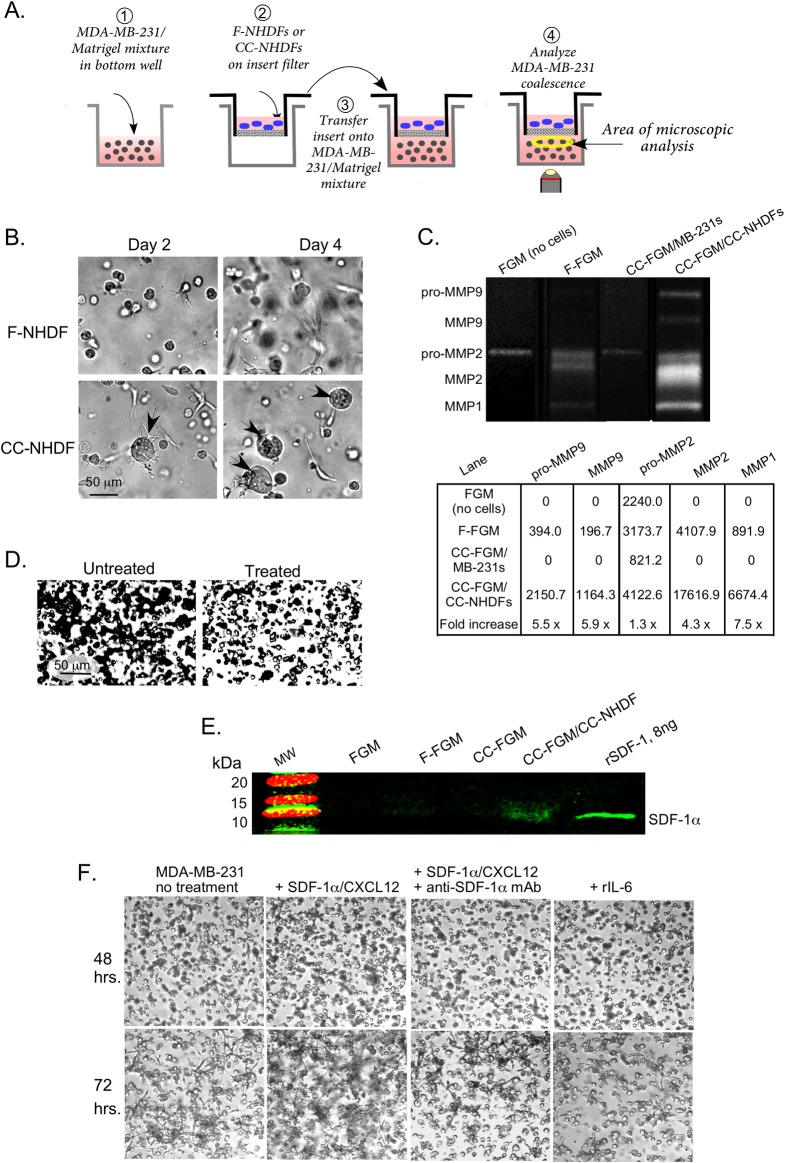
CC-NHDFs release soluble signals that accelerate aggregation and coalescence. A. The transmembrane preparation employed to assess the presence of soluble factors. B. Representative images of aggregation of MDA-MB-231 cells after 2 and 4 days in a preparation with F-NHDFs or CC-NHDFs in the upper insert. C. Gel zymogram and quantitation of pixel intensities of MMPs in FGM directly from the bottle of sterile, unused media (no cells); F-FGM, media from 72 hour cultures of F-NHDFs; CC-FGM/MB-231, media from 72 hour MDA-MB-231 cultures; and CC-FGM/CC-NHDF, media from 72 hour CC-NHDF. D. Aggregation after 48 hours of MDA-MB-231 preparations over a CC-NHDF substratum in the absence and presence of the MMP inhibitor GM6001. E. Western blot of media probed with anti-SDF-1α/CXCL12 mAb. Recombinant SDF-1α/CXCL12 was used as a positive control. F. Coalescence after 48 and 72 hours of untreated MDA-MB-231, MDA-MB-231 cells treated with SDF-1α/CXCL12, MDA-MB-231 cells treated with SDF-1α/CXCL12 plus anti-SDF-1α/CXCL12 mAb, and MDA-MB-231 cells treated with rIL-6.

To test whether CC-NHDFs also release soluble acceleration factors, MDA-MB-231/Matrigel mixtures were cast on the bottom wells in 24 well tissue culture dishes with removable filter inserts. The inserts were overlaid with a suspension of either F-NHDFs or CC-NHDFs and placed over the MDA-MB-231/Matrigel phase, the latter in the bottom wells (Fig 7A). The MDA-MB-231 cells that were separated from untreated F-NHDF cells underwent coalescence at a far slower rate than MDA-MB-231 cells separated from CC-NHDF cells (Fig 7B) and the aggregates were more spheroid than those formed on CC-NHDF scaffolds. Microscopic scanning revealed that no CC-NHDF cells or lamellipodia had traversed the micropore filter. The transwell experiment therefore demonstrated that, in addition to direct physical interaction, activated fibroblasts also stimulated coalescence of MDA-MB-231 cells by releasing soluble factors.

One possible candidate for acceleration factors released by activated fibroblasts were the MMPs [31]. The levels of pro-MMP9, MMP9, MMP2 and MMP1, assessed by zymography were 5.5, 5.9, 1.3 and 7.5 fold higher, respectively, in CC-FGM taken after 72 hours on CC-NHDF cells (lane CC-FGM/CC-NHDF) (Fig 7C) than in F-FGM taken after 72 hours on NHDF cells (lane F-FGM). A small amount of pro-MMP2 was present in all samples of FGM, but apart from that weak band, MDA-MB-231 cells did not release detectable levels of MMPs into the CC-FGM (lane CC-FGM/MB-231s) (Fig 7C). Thus, dermal fibroblasts secreted relatively high levels of MMPs in response to treatment with cancer cell conditioned media. To confirm these data, we tested whether the MMP inhibitor, GM6001, blocked acceleration of coalescence using the CC-NHDF cells. After 24 hours of incubation, MDA-MB-231 cells in Matrigel overlaying a CC-NHDF substratum had begun to aggregate, but MDA-MB-231 cells overlaid onto CC-NHDF cultures that had been treated with GM6001 remained randomly dispersed (Fig 7D).

The chemokine SDF-1α/CXCL12 is also released by activated fibroblasts [15] with reported effects on cancer cell locomotion and metastasis [32]. Our Western blot analysis confirmed that CC-NHDFs released SDF-1α/CXCL12 into the media at levels several fold higher than F-NHDFs or MDA-MB-231 cells (Fig 7E). We therefore grew MDA-MB-231 cells for 24 hours in the presence of SDF-1α/CXCL12 and monitored their subsequent aggregation and coalescence in the 3D Matrigel environment. Accelerated MDA-MB-231 cell aggregation and coalescence was noted after 48 hours and was quite evident after 72 hours (Fig 7F). Addition of the anti- SDF-1α/CXCL12 mAb to the culture medium containing SDF-1α/CXCL12 abrogated the effect (Fig 7F). Finally, we tested whether aggregation and coalescence were directly stimulated in MDA-MB-231 cells by treatment with rIL-6. To that end, rIL-6 was added to the growth medium for 24 hours prior to suspending the treated cells in Matrigel for the coalescence assay. Images acquired following 48 hours in Matrigel showed no effect from rIL-6 treatment of MDA-MB-231 cells, nor was acceleration observed at 72 hours (Fig 7F).

### Breast cancer cell-conditioned medium activates HPMFs affecting their polarity, morphology, gene expression, ability to accelerate coalescence and MMP release

**Fig 8:**
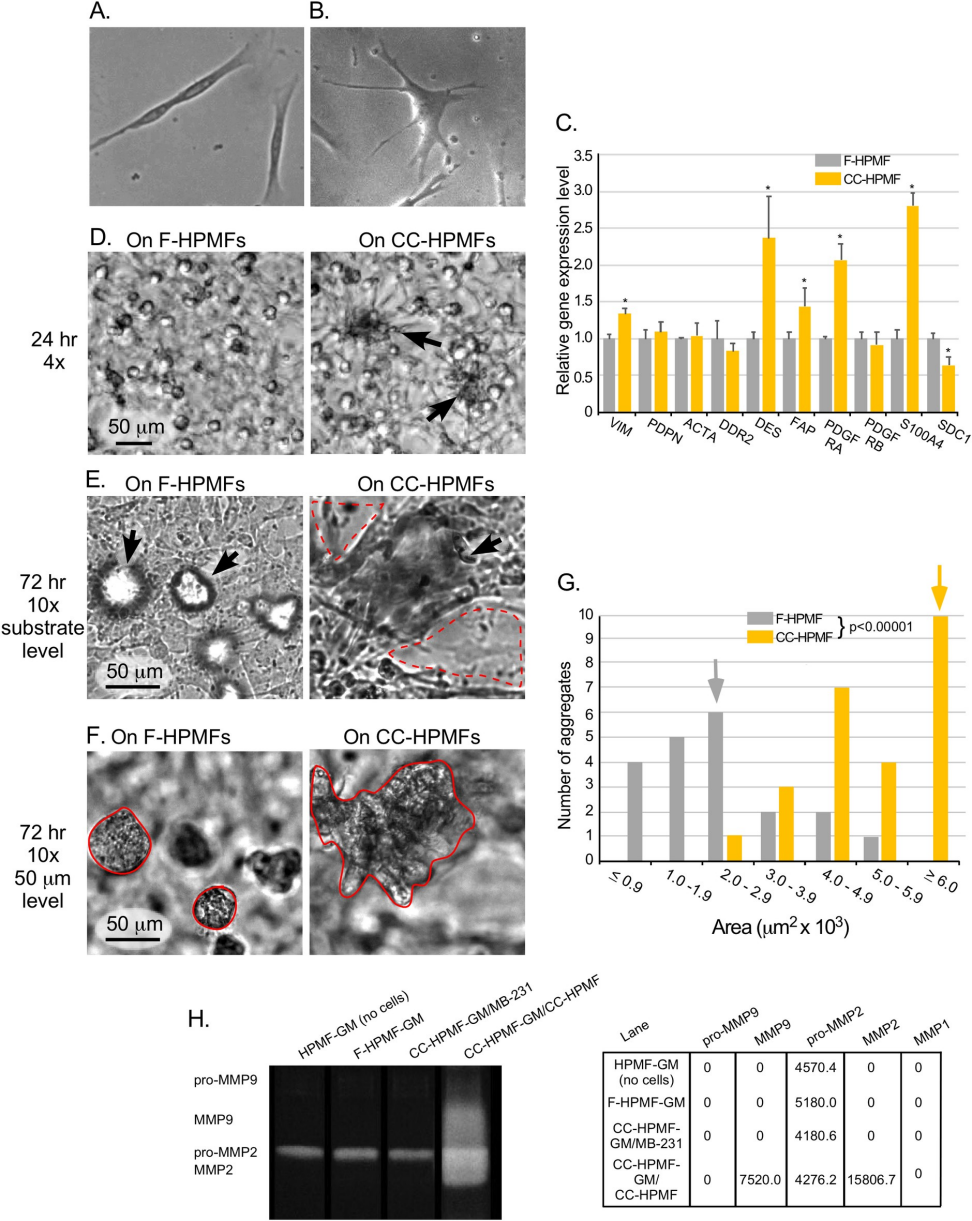
CC-HPMFs exhibit altered morphology, altered gene expression, accelerate MDA-MB-231 coalescence and release elevated MMPs. A, B. Representative phase images of F-HPMF and CC-HPMFs. C. qRT-PCR analyses of the transcript levels of ten genes associated with fibroblast activation. Means and error bars (standard deviations) are presented for N = 3. Asterisk (*) indicates significance of p <0.05. D. Representative fields after 24 hrs of aggregating MDA-MB-231 cells in which the MDA-MB-231/Matrigel phase is cast over F-HPMF cells on collagen and over CC-HPMF cells on collagen reveals larger aggregates in the latter. E. Substrate level, 10x magnification of representative fields of aggregating MDA-MB-231 cells over F-HPMF cells on collagen and over CC-HPMF cells on collagen reveals advanced coalescence and considerably more destruction of the CC-HPMF monolayer (dashed red line) in comparison to F-HPMF monolayer. F. Representative images of aggregating MDA-MB-231 cells cast over F-HPMF cells on collagen and over CC-HPMF cells on collagen at 72 hours at the 50 μm level demonstrate significantly larger aggregates over the CC-HPMF layer (solid red line) in comparison to the F-HPMF layer. G. Measurements of aggregate areas at 72 hours in cultures in which the MDA-MB-231/Matrigel phase was cast over F-HPMF cells (gray), and CC-HPMF cells (yellow). The median area of each population is noted as a gray or yellow arrow, respectively. The difference between MDA-MB-231 aggregate areas was significantly greater (p< 0.00001) in the presence of CC-HPMFs when compared to F-HPMFs. H. Gel zymogram and quantitation of pixel intensities of MMPs in HPMF-GM directly from the bottle of sterile, unused media (no cells); F-HPMF-GM, media from 72 hour cultures of F-HPMFs; CC-HPMF-GM/MB-231, media from 72 hour MDA-MB-231 cultures; and CC-HPMF-GM/CC-HPMF, media from 72 hour CC-HPMF cultures.

In order to confirm the results obtained with CC-NHDFs, we performed similar experiments with conditioned and unconditioned primary mammary fibroblasts. As was the case with the dermal fibroblasts, dramatic changes in morphologies were noted in CC-HPMFs on a collagen substrate. That is, while F-HPMFs were spindle shaped and bipolar (Fig 8A), similar to F-NHDF cells and consistent with the morphology of young, non-senescent fibroblasts [33], CC-HPMFs were spread and multipolar (Fig 8B). Next, to determine if factors released by MDA-MB-231 cells altered gene expression in CC-HPMFs, we examined the expression of the same ten genes by qRT-PCR that we assessed in the dermal fibroblasts. Interestingly, a different set of genes were upregulated in CC-HPMFs when compared to CC-NHDF cells. These included VIM, DES, FAP, PDFGRA and S100A4 while syndecan I (SDC1) was significantly downregulated (Fig 8 C). Nevertheless, when cast over CC-HPMFs, the rate of coalescence of MDA-MB-231 cells far exceeded that observed with CC-NHDFs and was evident as early as 24 hours (Fig 8 D, arrows). In addition, destruction of the CC-HPMF monolayer was even more pronounced (Fig 8E, dashed red line) and the area of aggregates appeared significantly greater (Fig 8F, solid red line), an observation that was confirmed by measuring aggregate areas (Fig 8G). To determine if CC-HPMFs, like CC-NHDFs, released elevated MMPs, pro-MMP9, MMP9, pro-MMP2, MMP2 and MMP1 were assessed by gel zymography (Fig 8G). Pro-MMP9 was not detectable in any of the HPMF-GM preparations while pro-MMP2 was present in all samples. MMP9 and MMP2 were only detectable in CC-HPMF-GM/CC-HPMF while MMP1 was not detected in any of the media. Therefore, although dermal and mammary fibroblasts release different collagenase enzymes of the MMP family, both release relatively high levels of MMPs in response to treatment with cancer cell conditioned media.

To determine if components specific to the HPMF-GM were stimulating the MDA-MB-231 cells to secrete activating factors into the conditioned medium, we performed the coalescence assay with different varieties of media. First, in a reciprocal media experiment, we conditioned HPMFs with MCF media [21] from 72 hour MDA-MB-231 (CC-MCF). Second, we prepared a minimal MCF medium (CC-minimal MCF) by omitting growth factors and supplementing with serum that had been stripped of growth factors. We then conditioned HPMFs with CC- minimal MCF from 72 hour MDA-MB-231 cells. As controls, we cultured HPMFs in unconditioned MCF and unconditioned minimal MCF. Again, aggregation and coalescence of MDA-MB-231 cells were accelerated in the presence of conditioned HPMFs as compared to their unconditioned counterparts with obvious destruction of the fibroblast monolayer, similar to observations in CC-HPMF, with a slight delay at 24 hours in the CC- minimal MCF (S2 Fig). Therefore, secretion of fibroblast stimulatory factors by MDA-MB-231 cells is not media specific, although serum associated cytokines and growth factors, prevalent in the tumor microenvironment [2, 34, 35], likely enhance their ability to secrete these stimulatory factors.

## Discussion

**Fig 9:**
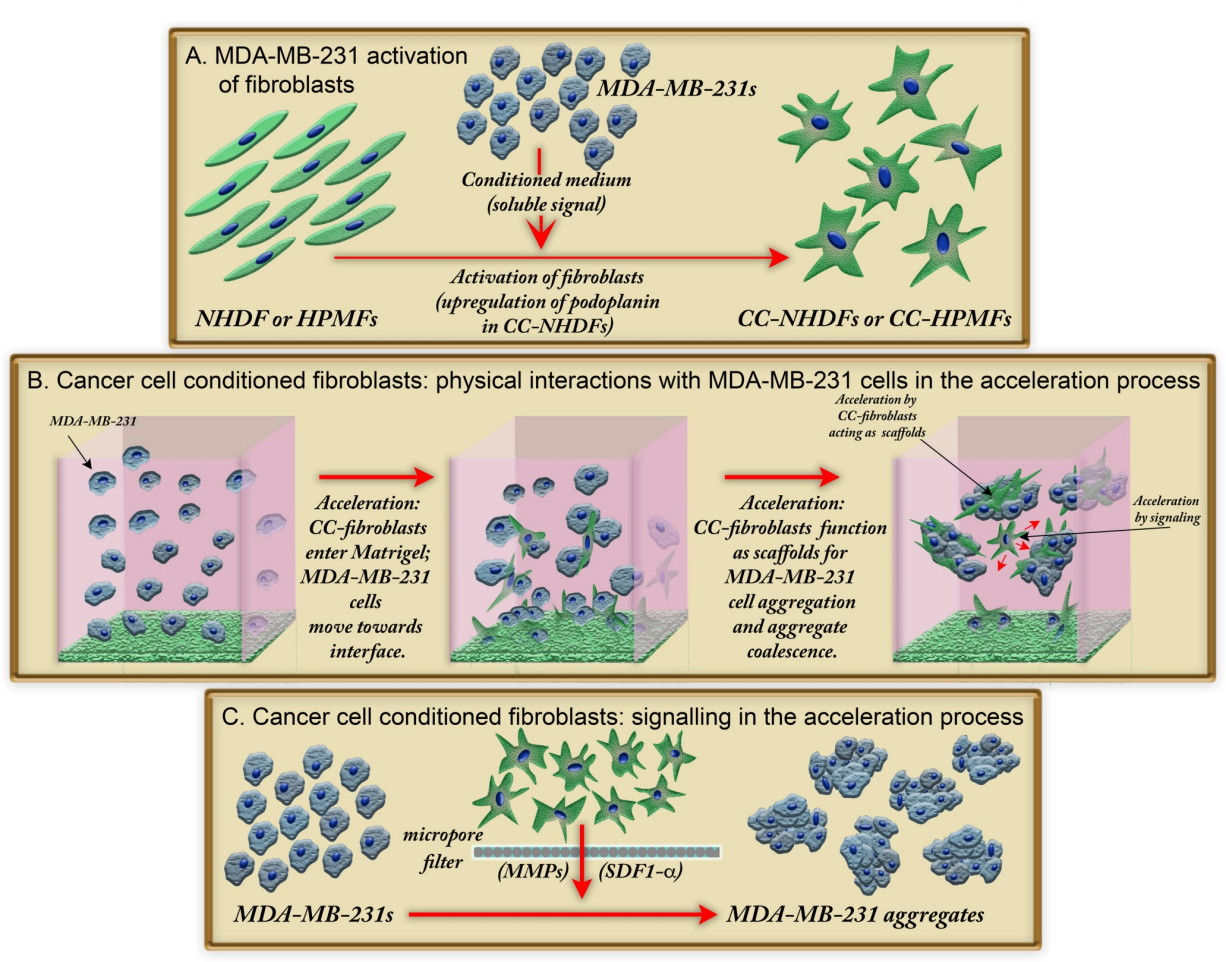
A model showing how activation of fibroblasts leads to accelerated coalescence of MDA-MB-231 breast cancer cells in a 3D Matrigel environment. A. Activation of NHDFs and HPMFs to CC-NHDFs and CC-HPMFs, respectively, after exposure to a medium conditioned by MDA-MB-231 cells. B. Physical interactions between MDA-MB-231 cancer cells and CC-fibroblasts occur initially at the collagen 1/Matrigel interface but later throughout the 3D environment with CC-fibroblasts serving as scaffolds for MDA-MB-231 cell coalescence. C. Release of soluble signals by CC-NHDF and CC-HPMF cells accelerates coalescence of MDA-MB-231 cells.

Both *in vitro* and *in vivo* studies have demonstrated that CAFs and tumor cells interact to drive tumor progression [12, 31], leading to several proposed models for reciprocal signaling [8, 36, 37]. Given the significance of reciprocal signaling and the formation of tumors in a 3D environment, we have developed a 3D model to analyze not only signaling, but also physical interactions between fibroblasts and cancer cells. We found that MDA-MB-231 cells release a fibroblast activation signal, and that these activated fibroblasts (CC-NHDFs and CC-HPMFs) then accelerate breast cancer cell aggregation and aggregate coalescence by, in turn, releasing soluble factors and also by serving as physical scaffolds for breast cancer cell aggregation (Fig 9). The soluble factors appear to include matrix metalloproteinases (MMPs) and, in the case of CC-NHDFs, the chemokine SDF-1α/CXCL12.

### NHDF activation

Fibroblasts can be activated by a number of molecular signals, depending upon body location, developmental circumstances such as embryogenesis, tissue maintenance, wound healing and tumorigenesis [8, 38–40]. Here, we have demonstrated that medium conditioned by the breast cancer cell line MDA-MB-231 activates NHDFs and HPMFs, transforming them into CAFs with altered morphology, motility and gene expression, most notably, the capacity to accelerate breast tumor-derived MDA-MB-231 cell aggregation and aggregate coalescence in a 3D environment.

NHDF activation by MDA-MB-231 conditioned medium resulted in more than a two-fold increase in the expression of five of the ten tested genes, selected for their association with fibroblast activation [2]. The five included PDPN, ACTA, DDR2, FAP and PDGFRB. Because PDPN and FAP were the most dramatically upregulated and also previously implicated in fibroblast activation [41], we tested whether overexpression of either imbued NHDFs with the capacity to accelerate MDA-MB-231 aggregation and coalescence. Overexpressing PDPN did imbue NHDFs with the capacity to accelerate, but overexpressing FAP did not. In addition, pretreatment of CC-NHDFs with anti-PDPN antibody blocked their capacity to accelerate. These results indicate that PDPN plays a central role in NHDF activation.

PDPN has been shown to regulate the Wnt/ß-cat signaling pathway, presumably through its interaction with ezrin/radixin/moesin (ERM) family proteins that link cytoskeletal structures to cell membranes [42]. Because PDPN was undetectable in NHDF cells, we assume that the activating factor released by MDA-MB-231 cells does not function through interaction with PDPN, but rather another receptor, and that activation through the Wnt/ß-cat pathway [42] results in *PDPN* upregulation.

Suchanski et al (2017) [41] found that PDPN overexpression in fibroblasts co-cultured with cancer cells did not enhance migration or invasion of cancer cells, but did increase the motility of fibroblasts, measured indirectly by the ability of activated fibroblasts to traverse a filter with an 8 μm pore size. Although we did not see increased velocity in our direct measurements of CC-NHDF cell motility in which PDPN was upregulated, our finding that activation causes shape changes and Matrigel invasion are consistent with their reported increased ability to penetrate a filter [41].

Activation of HPMFs resulted in upregulation of FAP, VIM, DES, PDFGRA and S100A4, while, interestingly, SCDI was significantly downregulated. In contrast to our results, in a study by Liakou et al (2016) [3], SCD1 was upregulated in HPMFs treated for 24 hours with media conditioned by MDA-MB-231 cells [3]. However, we employed a different conditioning protocol and, perhaps more importantly, our conditioning period was considerably longer. Thus, the discrepancy in SCD1 expression may be attributable to fibroblast plasticity and the existence of transitional fibroblast states. The difference is quite likely due to time-dependent changes in gene expression, including transcription factor genes, in a program of differentiation. CAFs are remarkably heterogeneic [2, 43] and transitional states [2, 34] with overlapping gene expression patterns are common in the tumor stroma [44]. The upregulation of VIM, DES, and FAP are features of myofibroblasts [45, 46]. Hence, it is reasonable to conclude that the longer conditioning period we employed may reflect a myofibroblast-like transitional state, and is consistent with the prevalence of stromal cells with myofibroblast characteristics in epithelial tumors [45].

### Acceleration by physical interactions

Although numerous studies have focused on diffusible, soluble factors released by CAFs, that promote cancer progression through EMT [18, 47, 48] and the concomitant acquisition of cancer cell motility and metastasis [2, 49, 50], only a few have examined possible physical interactions between CAFs and cancer cells. Gaggioli et al (2007) [10] showed that CAFs generated tracks in a Matrigel/collagen matrix into which overlying squamous cell carcinoma cells (SCCs) collectively migrated. When CAFs and SCCs were mixed, the leading cell was typically a fibroblast during collective migration of the SCCs. Pretreatment of the matrix with CAFs, followed by removal of CAFs and subsequent seeding of the SCC cells enabled SCC invasion, suggesting that during metastasis, CAFs generated tracks in the matrix that cancer cells subsequently entered. In a later study [11] it was reported that when CAFs and SCCs were co-cultured as spheroids in a Matrigel/collagen matrix, SCC cells followed the CAFs as the cells exited the spheroids. Most relevant to the results presented here, Knuchel et al (2015) [9] demonstrated that fibroblasts promote colorectal cancer (CRC) cell elongation and motility through direct cell-cell interactions, and that adhesion appears to be mediated by FGF-2, FGFR and integrin α_v_ß_5_. They also demonstrated that fibroblasts directed CRC cells to exit established CRC spheroids.

Our experimental design allowed us to observe dynamic physical interactions between fibroblasts overlayed with cancer cells in a 3D environment in which each cell type was plated in its normal microenvironment; i.e. fibroblasts on collagen [51] and cancer cells of epithelial origin in the basement membrane matrix Matrigel [52]. Our 4D analysis of live cultures revealed that fibroblasts were able to penetrate the matrix, and by 48 hours, functioned as scaffolds for cancer cell aggregation. Movement of MDA-MB-231 cells was directional, towards and along the elongated CC-NHDFs in the process of aggregation. Aggregates enveloped the CC-NHDFs. CC-NHDF migration and scaffolding occurred as much as 70 μm above the original CC-NHDF/Matrigel boundary. In support of our findings, fibroblasts isolated from the interface zone between breast tumors and stroma actively modulated tumor cell behavior in co-cultures to a greater extent than normal fibroblasts or fibroblasts within the tumor [53]. Importantly, the interactions revealed in our model might occur very early in *in vivo* tumorigenesis and may explain tumor growth and tumor heterogeneity in cases of field cancerization [19, 20, 54], a widely documented occurrence in many types of cancers [55–57], including breast cancer [54, 58], in which discrete, multiple islands or foci of neoplastic cells are present within the diseased tissue [59–61]. Based on an examination of 783 oral tumors, Slaughter [19] suggested that these foci grow independently and eventually coalesce to form a tumor, a suggestion that has been more recently documented in histological analyses of melanoma development in humans [62].

### Acceleration of coalescence by soluble factors released by CC-NHDF cells

CC-NHDFs accelerated aggregation and coalescence across the filter in a transwell assay without penetrating the filter, demonstrating that the CC-NHDFs released one or more soluble acceleration factors. However, the aggregates induced by soluble factors alone were far more compact than the aggregates accelerated by CC-NHDF scaffolding. This suggests either that the mechanism of acceleration by soluble factors differs from that mediated by physical scaffolding, or that in the latter case, the CC-NHDFs caused larger but more diffuse aggregates because their presence interfered with compactness of MDA-MB-231 cells. To identify acceleration factors, we tested several molecules reported to be secreted by activated fibroblasts. First, we found that CC-NHDFs and CC-HPMFs release matrix metalloproteinases (MMPs) which are released by activated fibroblasts [6] and remodel ECM [52], at five to ten times the level of inactivated fibroblasts, and that the MMP inhibitor GM6001 retarded aggregation and coalescence. MMPs, by remodeling the ECM, may facilitate fibroblast and MDA-MB-231 translocation through the 3D matrix, thus accelerating the process of aggregation and coalescence. Secondly, we found that the cytokine SDF-1α/CXCL12 is released by CC-NHDFs and accelerates aggregation and coalescence. SDF-1α/CXCL12, which has been demonstrated to affect cancer cell proliferation [63, 64] and metastasis [15], must directly affect MDA-MB-231 behavior, probably through the SDF-1α/CXCL12 receptor, which has been shown to be expressed in MDA-MB-231 cells [65].

## Conclusion

Based on the tumor cell-specific characteristics of cell aggregation and aggregate coalescence in a 3D environment, we designed an assay to investigate bidirectional signaling and physical cell-cell interactions of fibroblasts and breast tumor-derived MDA-MB-231 cells. We first demonstrated that tumor cells activated fibroblasts by releasing an unidentified factor. Activation appeared to involve up-regulation of podoplanin in dermal fibroblasts, which plays a major role in the effects these fibroblasts have on tumor cells. In turn, activated fibroblasts released one or more soluble signals, tentatively identified as matrix metalloproteinases and the cytokine SDF-1α/CXCL12, which accelerated the rate of tumor cell aggregation and aggregate coalescence by four-fold. HPMF activation involved a different gene expression pattern but nevertheless, similar to CC-NHDFs, MMP secretion was markedly increased along with the rate and extent of coalescence in breast cancer cells. We further demonstrated that activated fibroblasts also accelerate aggregation and coalescence by traversing a 3D Matrigel environment and physically acting as scaffolds for cancer cells. We hypothesize that the reciprocal signaling system, in conjunction with the direct interaction of activated fibroblasts with cancer cells, as revealed in the 3D Matrigel environment and described here, may reflect interactions between breast cancer cells and stromal fibroblasts *in vivo*. These interactions may ultimately facilitate tumor growth and coalescence in early stages of tumorigenesis in field cancerized tissue and may also reflect cancer cell-fibroblast interactions that do not involve coalescence during tumor development.

## Supporting information

S1 Table Primers used for qRT-PCR and plasmid construction in this study. (PDF)

S1 Fig Overexpression of FAP in NHDFs does not imbue NHDFs with the capacity to accelerate coalescence of MDA-MB-231 cells. (TIF)

S2 Fig Coalescence of MDA-MB-231 breast cancer cells is accelerated and is robust in the presence of CC-HPMF cells conditioned in CC-MCF and CC-minimal MCF media. (TIF)

## References

[1] Spaw M, Anant S, Thomas SM. Stromal contributions to the carcinogenic process. Molecular carcinogenesis. 2017;56(4):1199–213. 10.1002/mc.22583 27787930 PMC5354948

[2] Kalluri R. The biology and function of fibroblasts in cancer. Nat Rev Cancer. 2016;16(9):582–98. 10.1038/nrc.2016.73 27550820

[3] Liakou E, Mavrogonatou E, Pratsinis H, Rizou S, Evangelou K, Panagiotou PN, et al Ionizing radiation-mediated premature senescence and paracrine interactions with cancer cells enhance the expression of syndecan 1 in human breast stromal fibroblasts: the role of TGF-beta. Aging. 2016;8(8):1650–69. Epub 2016/07/20. 10.18632/aging.100989 27434331 PMC5032688

[4] Yang N, Mosher R, Seo S, Beebe D, Friedl A. Syndecan-1 in breast cancer stroma fibroblasts regulates extracellular matrix fiber organization and carcinoma cell motility. The American journal of pathology. 2011;178(1):325–35. Epub 2011/01/13. 10.1016/j.ajpath.2010.11.039 21224069 PMC3069862

[5] Levental KR, Yu H, Kass L, Lakins JN, Egeblad M, Erler JT, et al Matrix crosslinking forces tumor progression by enhancing integrin signaling. Cell. 2009;139(5):891–906. Epub 2009/11/26. 10.1016/j.cell.2009.10.027 19931152 PMC2788004

[6] Roy R, Yang J, Moses MA. Matrix metalloproteinases as novel biomarkers and potential therapeutic targets in human cancer. Journal of clinical oncology: official journal of the American Society of Clinical Oncology. 2009;27(31):5287–97. Epub 2009/09/10. 10.1200/jco.2009.23.5556 19738110 PMC2773480

[7] Soon PSH, Kim E, Pon CK, Gill AJ, Moore K, Spillane AJ, et al Breast cancer-associated fibroblasts induce epithelial-to-mesenchymal transition in breast cancer cells. Endocrine-Related Cancer. 2013;20(1):1–12. 10.1530/ERC-12-0227 23111755

[8] Gascard P, Tlsty TD. Carcinoma-associated fibroblasts: orchestrating the composition of malignancy. Genes Dev. 2016;30(9):1002–19. Epub 2016/05/07. 10.1101/gad.279737.116 27151975 PMC4863733

[9] Knuchel S, Anderle P, Werfelli P, Diamantis E, Rüegg C. Fibroblast surface-associated FGF-2 promotes contact-dependent colorectal cancer cell migration and invasion through FGFR-SRC signaling and integrin α(v)β(5)-mediated adhesion. Oncotarget. 2015;6(16):14300–17. PMC4546468. 10.18632/oncotarget.3883 25973543 PMC4546468

[10] Gaggioli C, Hooper S, Hidalgo-Carcedo C, Grosse R, Marshall JF, Harrington K, et al Fibroblast-led collective invasion of carcinoma cells with differing roles for RhoGTPases in leading and following cells. Nat Cell Biol. 2007;9(12):1392–400. Epub 2007/11/27. 10.1038/ncb1658 . 18037882

[11] Labernadie A, Kato T, Brugues A, Serra-Picamal X, Derzsi S, Arwert E, et al A mechanically active heterotypic E-cadherin/N-cadherin adhesion enables fibroblasts to drive cancer cell invasion. Nat Cell Biol. 2017;19(3):224–37. 10.1038/ncb3478 http://www.nature.com/ncb/journal/v19/n3/abs/ncb3478.html#supplementary-information. 28218910 PMC5831988

[12] Giannoni E, Bianchini F, Masieri L, Serni S, Torre E, Calorini L, et al Reciprocal activation of prostate cancer cells and cancer-associated fibroblasts stimulates epithelial-mesenchymal transition and cancer stemness. Cancer Res. 2010;70(17):6945–56. Epub 2010/08/12. 10.1158/0008-5472.CAN-10-0785 . 20699369

[13] Scherer A, Kuhl S, Wessels D, Lusche DF, Hanson B, Ambrose J, et al A Computer-Assisted 3D Model for Analyzing the Aggregation of Tumorigenic Cells Reveals Specialized Behaviors and Unique Cell Types that Facilitate Aggregate Coalescence. PloS one. 2015;10(3):e0118628 10.1371/journal.pone.0118628 25790299 PMC4366230

[14] Wessels D, Lusche DF, Voss E, Kuhl S, Buchele EC, Klemme MR, et al Melanoma cells undergo aggressive coalescence in a 3D Matrigel model that is repressed by anti-CD44. PloS one. 2017;12(3):e0173400 Epub 2017/03/07. 10.1371/journal.pone.0173400 28264026 PMC5338862

[15] Ahirwar DK, Nasser MW, Ouseph MM, Elbaz M, Cuitiño MC, Kladney RD, et al Fibroblast-derived CXCL12 promotes breast cancer metastasis by facilitating tumor cell intravasation. Oncogene. 2018 10.1038/s41388-018-0263-7 29720724 PMC7063845

[16] Attieh Y, Vignjevic DM. The hallmarks of CAFs in cancer invasion. European journal of cell biology. 2016;95(11):493–502. Epub 2016/08/31. 10.1016/j.ejcb.2016.07.004 . 27575401

[17] Glentis A, Oertle P, Mariani P, Chikina A, El Marjou F, Attieh Y, et al Cancer-associated fibroblasts induce metalloprotease-independent cancer cell invasion of the basement membrane. Nature Communications. 2017;8(1):924 10.1038/s41467-017-00985-8 29030636 PMC5640679

[18] Tao L, Huang G, Song H, Chen Y, Chen L. Cancer associated fibroblasts: An essential role in the tumor microenvironment. Oncology letters. 2017;14(3):2611–20. Epub 2017/09/21. 10.3892/ol.2017.6497 28927027 PMC5588104

[19] Slaughter DP, Southwick HW, Smejkal W. Field cancerization in oral stratified squamous epithelium; clinical implications of multicentric origin. Cancer. 1953;6(5):963–8. Epub 1953/09/01. . 13094644 10.1002/1097-0142(195309)6:5<963::aid-cncr2820060515>3.0.co;2-q

[20] Liao Z, Tan ZW, Zhu P, Tan NS. Cancer-associated fibroblasts in tumor microenvironment–Accomplices in tumor malignancy. Cellular Immunology. 2018 10.1016/j.cellimm.2017.12.003. 29397066

[21] Lusche DF, Buchele EC, Russell KB, Soll BA, Vitolo MI, Klemme MR, et al Overexpressing TPTE2 (TPIP), a homolog of the human tumor suppressor gene PTEN, rescues the abnormal phenotype of the PTEN(-/-) mutant. Oncotarget. 2018;9(30):21100–21. Epub 2018/05/17. 10.18632/oncotarget.24941 29765523 PMC5940379

[22] Ambrose J, Livitz M, Wessels D, Kuhl S, Lusche DF, Scherer A, et al Mediated coalescence: a possible mechanism for tumor cellular heterogeneity. American journal of cancer research. 2015;5(11):3485–504. Epub 2016/01/26. 26807328 PMC4697694

[23] Kuhl S, Voss E, Scherer A, Lusche DF, Wessels D, Soll DR. 4D Tumorigenesis model for quantitating coalescence, quantitating directed cell motility and chemotaxis, identifying unique cell behaviors and testing anti-cancer drugs In: Hereld D, Jin T, editors. Chemotaxis: Methods and Protocols: Springer; 2016. 10.1007/978-1-4939-3480-5_18 27271907

[24] Soll D, Voss E. Two and three dimensional computer systems for analyzing how cells crawl In: Soll D, Wessels D, editors. Motion Analysis of Living Cells: John Wiley, Inc 1998 p. 25–52.

[25] Soll DR, Voss E, Wessels D, Kuhl S. Computer-Assisted Systems for Dynamic 3D Reconstruction and Motion Analysis of Living Cells In: Shorte S, Frischknecht F, editors. Imaging Cellular and Molecular Biological Functions. Principles and Practice: Springer Berlin Heidelberg; 2007 p. 365–84.

[26] Sanchez P, Daniels KJ, Park YN, Soll DR. Generating a battery of monoclonal antibodies against native green fluorescent protein for immunostaining, FACS, IP, and ChIP using a unique adjuvant. Monoclonal antibodies in immunodiagnosis and immunotherapy. 2014;33(2):80–8. Epub 2014/04/22. 10.1089/mab.2013.0089 24746148 PMC3998673

[27] Lusche DF, Klemme MR, Soll BA, Reis RJ, Forrest CC, Nop TS, et al Integrin α-3 ß-1’s central role in breast cancer, melanoma and glioblastoma cell aggregation revealed by antibodies with blocking activity. mAbs. 2019:null-null. 10.1080/19420862.2019.1583987 30810437 PMC6601557

[28] Schneider CA, Rasband WS, Eliceiri KW. NIH Image to ImageJ: 25 years of image analysis. Nat Methods. 2012;9(7):671–5. Epub 2012/08/30. 10.1038/nmeth.2089 22930834 PMC5554542

[29] Puré E, Lo A. Can targeting stroma pave the way to enhanced antitumor immunity and immunotherapy of solid tumors? Cancer immunology research. 2016;4(4):269–78. 10.1158/2326-6066.CIR-16-0011 PMC5452418. 27036971 PMC5452418

[30] Wessels DJ, Lusche DF, Kuhl S, Scherer A, Voss E, Soll DR. Quantitative Motion Analysis in Two and Three Dimensions. Methods in molecular biology (Clifton, NJ). 2016;1365:265–92. Epub 2015/10/27. 10.1007/978-1-4939-3124-8_14 . 26498790

[31] Elenbaas B, Weinberg RA. Heterotypic Signaling between Epithelial Tumor Cells and Fibroblasts in Carcinoma Formation. Experimental cell research. 2001;264(1):169–84. 10.1006/excr.2000.5133 11237532

[32] Kollmar O, Rupertus K, Scheuer C, Junker B, Tilton B, Schilling MK, et al Stromal Cell-Derived Factor-1 Promotes Cell Migration and Tumor Growth of Colorectal Metastasis. Neoplasia (New York, NY). 2007;9(10):862–70. PMC2040213. 10.1593/neo.07559 PMC2040213 17971906

[33] Papadopoulou A, Kletsas D. Human lung fibroblasts prematurely senescent after exposure to ionizing radiation enhance the growth of malignant lung epithelial cells in vitro and in vivo. Int J Oncol. 2011;39(4):989–99. Epub 2011/08/05. 10.3892/ijo.2011.1132 . 21814715

[34] Ohlund D, Elyada E, Tuveson D. Fibroblast heterogeneity in the cancer wound. J Exp Med. 2014;211 10.1084/jem.20140692 25071162 PMC4113948

[35] Bhowmick NA, Neilson EG, Moses HL. Stromal fibroblasts in cancer initiation and progression. Nature. 2004;432(7015):332–7. Epub 2004/11/19. 10.1038/nature03096 15549095 PMC3050735

[36] Cirri P, Chiarugi P. Cancer-associated-fibroblasts and tumour cells: a diabolic liaison driving cancer progression. Cancer Metastasis Rev. 2012;31(1–2):195–208. Epub 2011/11/22. 10.1007/s10555-011-9340-x . 22101652

[37] Hugo HJ, Lebret S, Tomaskovic-Crook E, Ahmed N, Blick T, Newgreen DF, et al Contribution of Fibroblast and Mast Cell (Afferent) and Tumor (Efferent) IL-6 Effects within the Tumor Microenvironment. Cancer Microenvironment. 2012;5(1):83–93. 10.1007/s12307-012-0098-7 22314376 PMC3343200

[38] Buckley CD, Pilling D, Lord JM, Akbar AN, Scheel-Toellner D, Salmon M. Fibroblasts regulate the switch from acute resolving to chronic persistent inflammation. Trends in immunology. 2001;22(4):199–204. Epub 2001/03/29. . 11274925 10.1016/s1471-4906(01)01863-4

[39] Chang HY, Chi J-T, Dudoit S, Bondre C, van de Rijn M, Botstein D, et al Diversity, topographic differentiation, and positional memory in human fibroblasts. Proceedings of the National Academy of Sciences. 2002;99(20):12877. 10.1073/pnas.162488599 PMC130553 12297622

[40] Darby IA, Laverdet B, Bonte F, Desmouliere A. Fibroblasts and myofibroblasts in wound healing. Clinical, cosmetic and investigational dermatology. 2014;7:301–11. Epub 2014/11/15. 10.2147/CCID.S50046 25395868 PMC4226391

[41] Suchanski J, Tejchman A, Zacharski M, Piotrowska A, Grzegrzolka J, Chodaczek G, et al Podoplanin increases the migration of human fibroblasts and affects the endothelial cell network formation: A possible role for cancer-associated fibroblasts in breast cancer progression. PloS one. 2017;12(9):e0184970 Epub 2017/09/25. 10.1371/journal.pone.0184970 28938000 PMC5609749

[42] Bresson L, Faraldo MM, Di-Cicco A, Quintanilla M, Glukhova MA, Deugnier MA. Podoplanin regulates mammary stem cell function and tumorigenesis by potentiating Wnt/beta-catenin signaling. Development (Cambridge, England). 2018;145(4). Epub 2018/01/24. 10.1242/dev.160382 . 29361573

[43] Yamauchi M, Barker TH, Gibbons DL, Kurie JM. The fibrotic tumor stroma. The Journal of Clinical Investigation. 2018;128(1):16–25. 10.1172/JCI93554 29293090 PMC5749516

[44] Busch S, Andersson D, Bom E, Walsh C, Ståhlberg A, Landberg G. Cellular organization and molecular differentiation model of breast cancer-associated fibroblasts. Molecular Cancer. 2017;16(1):73 10.1186/s12943-017-0642-7 28372546 PMC5376683

[45] Desmouliere A, Guyot C, Gabbiani G. The stroma reaction myofibroblast: a key player in the control of tumor cell behavior. The International journal of developmental biology. 2004;48(5–6):509–17. Epub 2004/09/07. 10.1387/ijdb.041802ad . 15349825

[46] Kuzet SE, Gaggioli C. Fibroblast activation in cancer: when seed fertilizes soil. Cell and tissue research. 2016;365(3):607–19. Epub 2016/07/31. 10.1007/s00441-016-2467-x . 27474009

[47] Gao MQ, Kim BG, Kang S. Stromal fibroblasts from the interface zone of human breast carcinomas induce an epithelial-mesenchymal transition-like state in breast cancer cells in vitro. Journal of cell science. 2010;123 10.1242/jcs.072900 20841377

[48] Zeisberg M, Neilson EG. Biomarkers for epithelial-mesenchymal transitions. J Clin Invest. 2009;119(6):1429–37. Epub 2009/06/03. 10.1172/JCI36183 19487819 PMC2689132

[49] Buchsbaum RJ, Oh SY. Breast Cancer-Associated Fibroblasts: Where We Are and Where We Need to Go. Cancers. 2016;8(2). Epub 2016/02/02. 10.3390/cancers8020019 26828520 PMC4773742

[50] Martin-Villar E, Fernandez-Munoz B, Parsons M, Yurrita MM, Megias D, Perez-Gomez E, et al Podoplanin associates with CD44 to promote directional cell migration. Molecular biology of the cell. 2010;21(24):4387–99. Epub 2010/10/22. 10.1091/mbc.E10-06-0489 20962267 PMC3002391

[51] Brouty-Boyé D. Developmental Biology of Fibroblasts and Neoplastic Disease In: Macieira-Coelho A, editor. Developmental Biology of Neoplastic Growth. Berlin, Heidelberg: Springer Berlin Heidelberg; 2005 p. 55–77. 10.1007/3-540-27671-8_3 17153480

[52] Kelley LC, Lohmer LL, Hagedorn EJ, Sherwood DR. Traversing the basement membrane in vivo: a diversity of strategies. The Journal of cell biology. 2014;204(3):291–302. Epub 2014/02/05. 10.1083/jcb.201311112 24493586 PMC3912525

[53] Gao MQ, Kim BG, Kang S, Choi YP, Park H, Kang KS, et al Stromal fibroblasts from the interface zone of human breast carcinomas induce an epithelial-mesenchymal transition-like state in breast cancer cells in vitro. Journal of cell science. 2010;123(Pt 20):3507–14. Epub 2010/09/16. 10.1242/jcs.072900 . 20841377

[54] Heaphy CM, Griffith JK, Bisoffi M. Mammary field cancerization: molecular evidence and clinical importance. Breast cancer research and treatment. 2009;118(2):229–39. Epub 2009/08/18. 10.1007/s10549-009-0504-0 . 19685287

[55] Franklin WA, Gazdar AF, Haney J, Wistuba II, La Rosa FG, Kennedy T, et al Widely dispersed p53 mutation in respiratory epithelium. A novel mechanism for field carcinogenesis. J Clin Invest. 1997;100(10):2417–639. Epub 1997/10/23. 10.1172/JCI119782 9329980 PMC508406

[56] Jones TD, Wang M, Eble JN, MacLennan GT, Lopez-Beltran A, Zhang S, et al Molecular evidence supporting field effect in urothelial carcinogenesis. Clinical cancer research: an official journal of the American Association for Cancer Research. 2005;11(18):6512–9. Epub 2005/09/17. 10.1158/1078-0432.ccr-05-0891 . 16166427

[57] Nonn L, Ananthanarayanan V, Gann PH. Evidence for field cancerization of the prostate. The Prostate. 2009;69(13):1470–9. Epub 2009/05/23. 10.1002/pros.20983 19462462 PMC3690597

[58] Lynch SP, Lei X, Hsu L, Meric-Bernstam F, Buchholz TA, Zhang H, et al Breast cancer multifocality and multicentricity and locoregional recurrence. Oncologist. 2013;18(11):1167–73. Epub 2013/10/19. 10.1634/theoncologist.2013-0167 24136008 PMC3825299

[59] Aung PP, Mutyambizi KK, Danialan R, Ivan D, Prieto VG. Differential diagnosis of heavily pigmented melanocytic lesions: challenges and diagnostic approach. Journal of clinical pathology. 2015;68(12):963–70. Epub 2015/11/26. 10.1136/jclinpath-2015-202887 . 26602414

[60] Chan JS, Tan MJ, Sng MK, Teo Z, Phua T, Choo CC, et al Cancer-associated fibroblasts enact field cancerization by promoting extratumoral oxidative stress. Cell death & disease. 2017;8(1):e2562 Epub 2017/01/20. 10.1038/cddis.2016.492 28102840 PMC5386391

[61] Costa S, Byrne M, Pissaloux D, Haddad V, Paindavoine S, Thomas L, et al Melanomas Associated With Blue Nevi or Mimicking Cellular Blue Nevi: Clinical, Pathologic, and Molecular Study of 11 Cases Displaying a High Frequency of GNA11 Mutations, BAP1 Expression Loss, and a Predilection for the Scalp. The American journal of surgical pathology. 2016;40(3):368–77. Epub 2015/12/10. 10.1097/PAS.0000000000000568 . 26645730

[62] Kutzner H, Metzler G, Argenyi Z, Requena L, Palmedo G, Mentzel T, et al Histological and genetic evidence for a variant of superficial spreading melanoma composed predominantly of large nests. Modern pathology: an official journal of the United States and Canadian Academy of Pathology, Inc. 2012;25(6):838–45. Epub 2012/03/06. 10.1038/modpathol.2012.35 . 22388759

[63] Addadi Y, Moskovits N, Granot D, Lozano G, Carmi Y, Apte RN, et al p53 status in stromal fibroblasts modulates tumor growth in an SDF1-dependent manner. Cancer Res. 2010;70(23):9650–8. Epub 2010/10/19. 10.1158/0008-5472.CAN-10-1146 20952507 PMC2999653

[64] Orimo A, Gupta PB, Sgroi DC, Arenzana-Seisdedos F, Delaunay T, Naeem R, et al Stromal fibroblasts present in invasive human breast carcinomas promote tumor growth and angiogenesis through elevated SDF-1/CXCL12 secretion. Cell. 2005;121(3):335–48. Epub 2005/05/11. 10.1016/j.cell.2005.02.034 . 15882617

[65] Wendt MK, Cooper AN, Dwinell MB. Epigenetic silencing of CXCL12 increases the metastatic potential of mammary carcinoma cells. Oncogene. 2007;27:1461 10.1038/sj.onc.1210751 https://www.nature.com/articles/1210751#supplementary-information. 17724466

