# Supplementary material for: Reciprocal signaling and direct physical interactions between fibroblasts and breast cancer cells in a 3D environment
Source: PLoS One. 2019 Jun 24;14(6):e0218854. doi: 10.1371/journal.pone.0218854 (PMC6590889; doi:10.1371/journal.pone.0218854)
Supplement: S1 Table — (PDF) [file pone.0218854.s001.pdf]

Supplemental Table S1. Primers used for qRT-PCR and plasmid construction in this study.

**Primer**	**Sequence (5´ to 3´)**
ACTA-f	GTGACGAAGCACAGAGCAAA
ACTA-r	CTTTTCCATGTCGTCCCAGT
DDR2-f	TTTGGGGAGGTTCATCTCTG
DDR2-r	ATTTTCACAGCCACCAGGAC
Desmin-f	AGGCAGCCAACAAGAACAAC
Desmin-r	GCCTCATCAGGGAATCGTTA
FAP-f	TTCATGATGGACGCACTGAT
FAP-r	TTCCTCTTTAGGCAGCTGGA
PDGFA-f	AGTCAGGGGAAACGATTGTG
PDGFA-r	TGCCTTTGCCTTTCACTTCT
PDGFRB-f	ACGAGAAGAAAGGGGACGTT
PDGFRB-r	CAATGGTGGTTTTGCAGATG
S100A4-f	AGCTTCTTGGGGAAAAGGAC
S100A4-r	TCTTGGAAGTCCACCTCGTT
Vim-f	CGAAAACACCCTGCAATCTT
Vim-r	ATTCCACTTTGCGTTCAAGG
podoplanin-f	CTGCTCTTCGTTTTGGGAAG
podoplanin-r	TTCCTGGAGTCACCACATCA
syndecan-f	TGCTGTACCGCATGAAGAAG
syndecan-r	GCATAGAATTCCTCCTGTTTGG
GAPDH-f	AATGACCCCTTCATTGACCTC
GAPDH-r	TGGAAGATGGTGATGGGATT
FAP-1	TATA<u>ACCGGT</u>ATGAAGACTTGGGTAAAAATCGTAT
FAP-2	ATAT<u>GTCGAC</u>CTAGTCTGACAAAGAGAAACACTGCTT
mCherry-1	TATA<u>ACCGGT</u>ATGGTTTCAAAAGGTGAAGAAG
mCherry-2	ATAT<u>AGATCT</u>TTTATATAATTCATCCATACC
PDPN-1	TATA<u>ACCGGT</u>ATGTGGAAGGTGTCAGCTCTGCTCTTC
PDPN-2	ATAT<u>GTCGAC</u>CTAGGGCGAGTACCTTCCCGACATTTTTCGC
