# Supplementary figures and images for: Reciprocal signaling and direct physical interactions between fibroblasts and breast cancer cells in a 3D environment
Source: PLoS One. 2019 Jun 24;14(6):e0218854. doi: 10.1371/journal.pone.0218854 (PMC6590889; doi:10.1371/journal.pone.0218854)

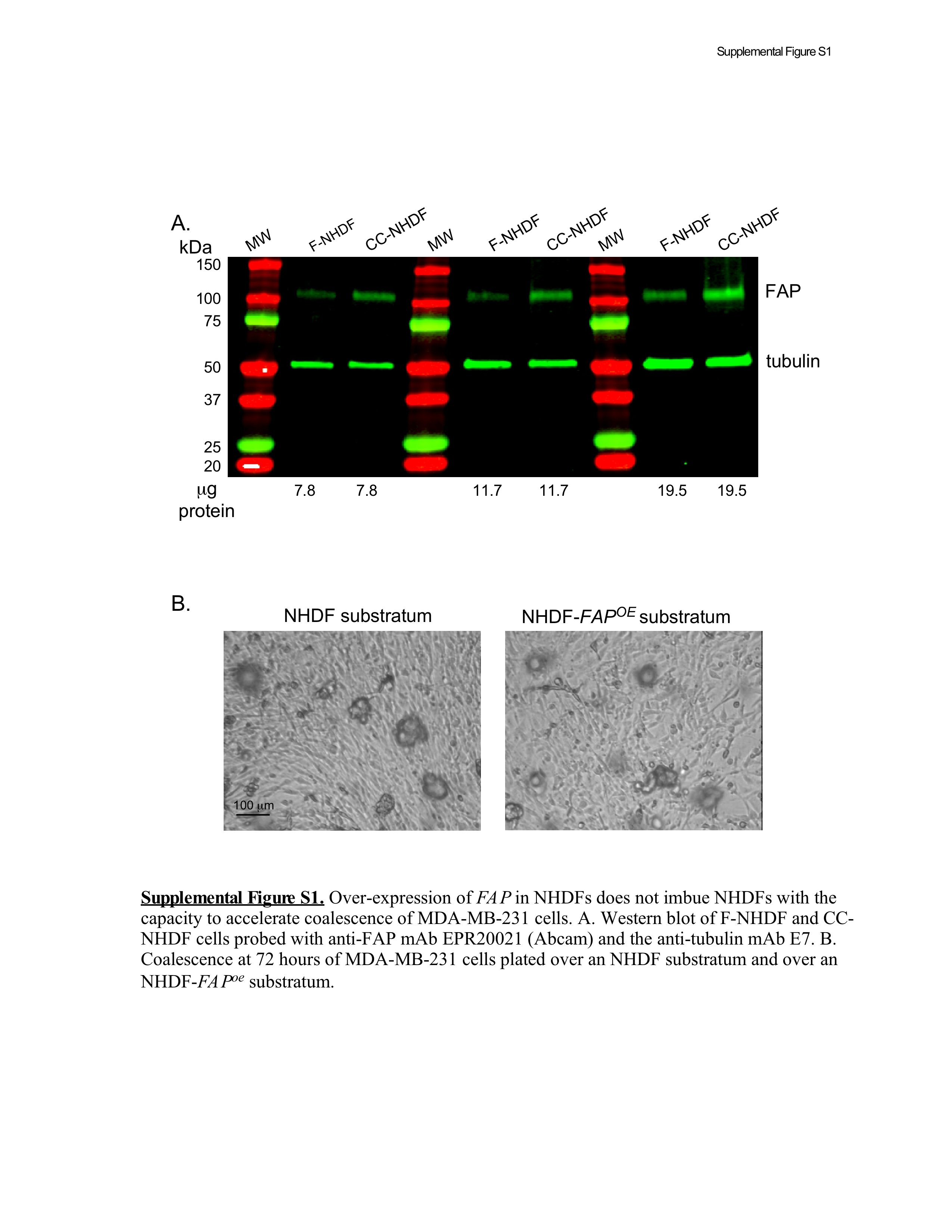

Supplement: S1 Fig — (TIF) [file pone.0218854.s002.tif]

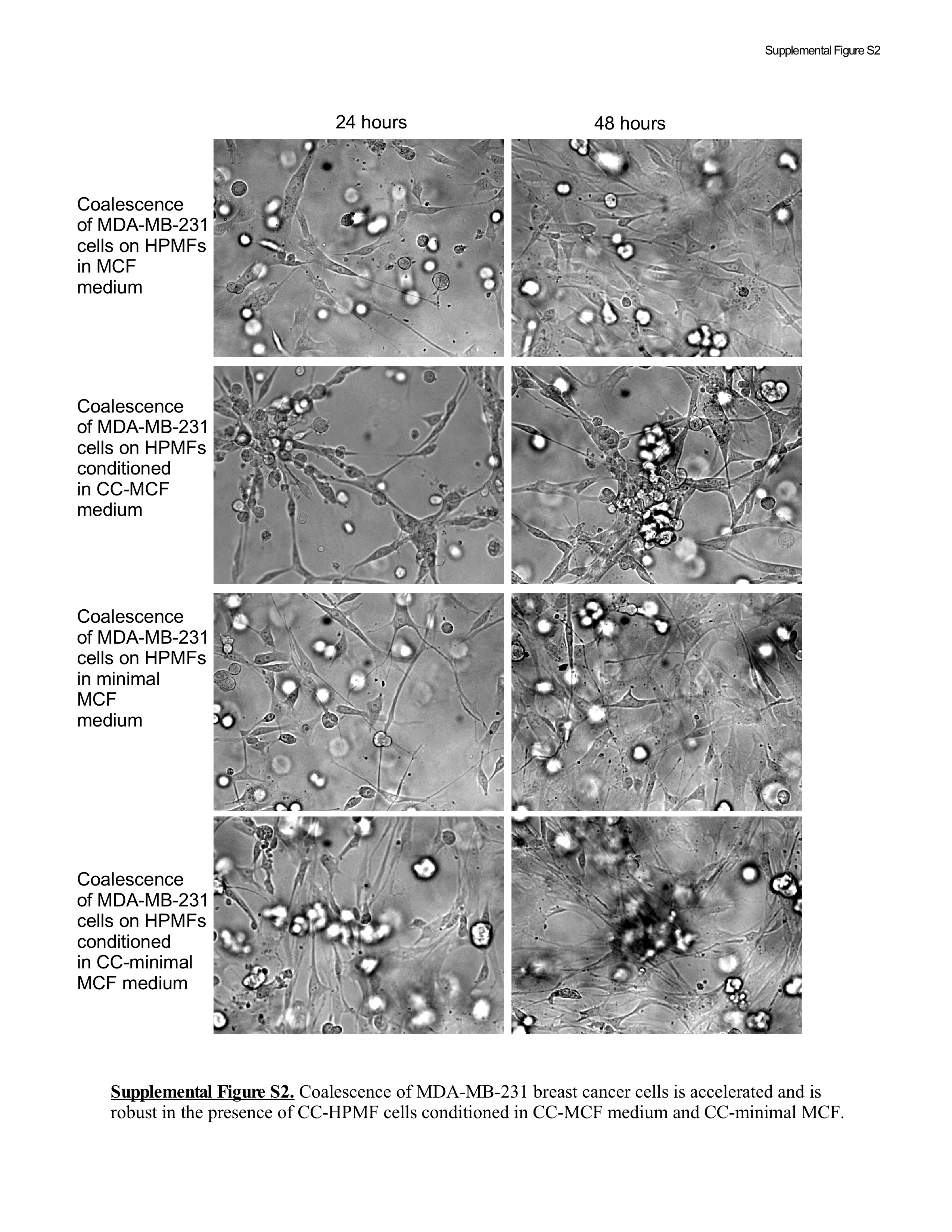

Supplement: S2 Fig — (TIF) [file pone.0218854.s003.tif]
